# HyperSTAR: Unveiling Tissue Structure and Tumor Microenvironment from Spatial Omics by Hypergraph Learning

**DOI:** 10.1093/gpbjnl/qzaf128

**Published:** 2025-12-26

**Authors:** Yi Liao, Chong Zhang, Zhikang Wang, Fei Qi, Weitian Huang, Shangyan Cai, Junyu Li, Jiazhou Chen, Robin B Gasser, Zhiyuan Yuan, Jiangning Song, Hongmin Cai

**Affiliations:** School of Computer Science and Engineering, South China University of Technology, Guangzhou 510006, China; School of Computer Science and Engineering, South China University of Technology, Guangzhou 510006, China; Biomedicine Discovery Institute and Department of Biochemistry and Molecular Biology, Monash University, Melbourne, VIC 3800, Australia; Monash AI Institute, Monash University, Melbourne, VIC 3800, Australia; School of Data Science and Information Engineering, Guizhou Minzu University, Guiyang 550025, China; School of Future Technology, South China University of Technology, Guangzhou 511442, China; Faculty of Science and Technology, Beijing Normal University-Hong Kong Baptist University United International College, Zhuhai 519087, China; School of Future Technology, South China University of Technology, Guangzhou 511442, China; School of Computer Science and Technology, Guangdong University of Technology, Guangzhou 510006, China; Department of Veterinary Biosciences, Melbourne Veterinary School, Faculty of Science, The University of Melbourne, Parkville, VIC 3010, Australia; Institute of Science and Technology for Brain-Inspired Intelligence, MOE Key Laboratory of Computational Neuroscience and Brain-Inspired Intelligence, MOE Frontiers Center for Brain Science, Fudan University, Shanghai 200433, China; Center for Medical Research and Innovation, Shanghai Pudong Hospital, Fudan University Pudong Medical Center, Fudan University, Shanghai 201399, China; Biomedicine Discovery Institute and Department of Biochemistry and Molecular Biology, Monash University, Melbourne, VIC 3800, Australia; Monash AI Institute, Monash University, Melbourne, VIC 3800, Australia; School of Computer Science and Engineering, South China University of Technology, Guangzhou 510006, China; School of Future Technology, South China University of Technology, Guangzhou 511442, China

**Keywords:** Spatial omics, Spatial domain, Resolution, Hypergraph learning, Higher-order relationship

## Abstract

Spatial omics technologies have revolutionized life sciences by enabling the simultaneous acquisition of biomolecular and spatial information. Identifying spatial patterns is crucial for understanding organ development and tumor microenvironments. However, the emergence of diverse spatial omics resolutions in these technologies has made it challenging to accurately characterize spatial domains at finer resolutions. To address this, we propose HyperSTAR, a hypergraph-based method designed to precisely identify spatial domains across varying resolutions by leveraging higher-order relationships among spatially adjacent tissue programs. Specifically, a gene expression-guided hyperedge decomposition module is introduced to refine the hypergraph structure to accurately delineate spatial domain boundaries. Additionally, a hypergraph attention convolutional neural network is designed to adaptively learn the importance of each hyperedge, enhancing the model’s ability to capture complex higher-order relationships within spatially neighboring multi-spots and/or single cells. HyperSTAR outperforms existing graph neural network models in tasks such as uncovering tissue substructures, inferring spatiotemporal patterns, and denoising spatially resolved gene expression. It effectively handles diverse spatial omics data types and scales seamlessly to large datasets. The method successfully reveals spatial heterogeneity in breast cancer sections, with findings validated through functional and survival analyses of independent clinical data. HyperSTAR represents a significant advancement in spatial omics analysis, representing a robust tool for exploring complex spatial patterns across varying resolutions and data types. Its ability to capture intricate higher-order relationships among spatially neighboring spots/cells makes it an invaluable tool for advancing research in life sciences, particularly in cancer and developmental biology. The toolbox is available at https://github.com/Ringoio/HyperSTAR.

## Graphical abstract

**Figure:**
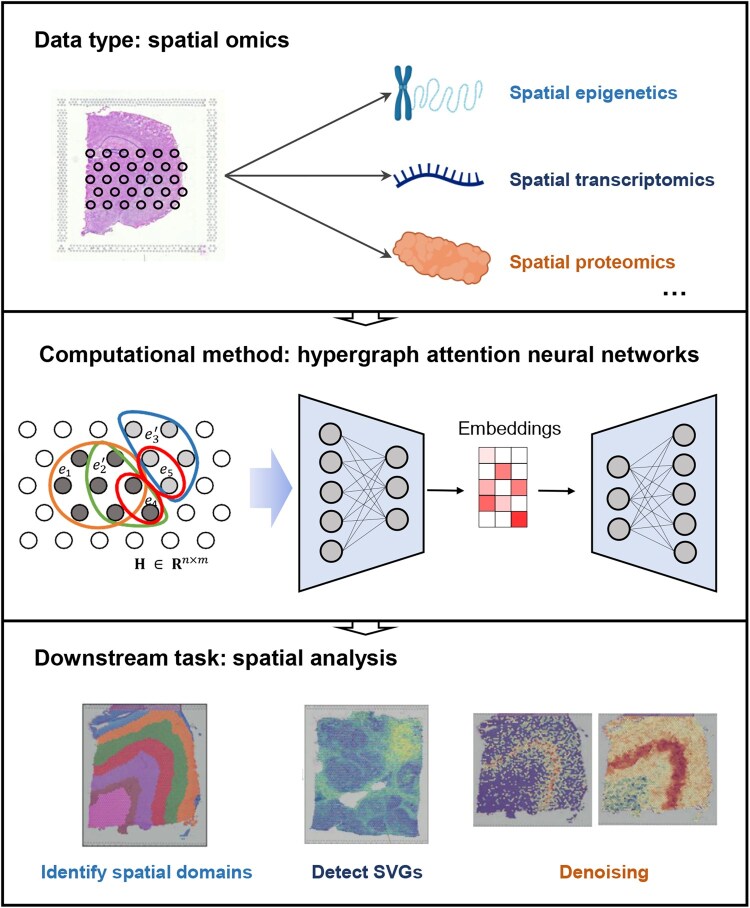

## Introduction

In living organisms, biological processes occur within a spatial context. The location of each cell is as crucial as its inherent characteristics in determining cell types, cell states, and functions [1]. Single-cell omics quantifies genomes, epigenomes, transcriptomes, and proteomes at the single-cell level [2,3]. Nevertheless, the process of single-cell dissociation during sample preparation results in the loss of spatial information [4]. Emerging spatially resolved transcriptomics (SRT) has facilitated the profiling of gene expression while preserving spatial location in tissues, offering valuable opportunities to explore intricate mechanisms within biological systems [5].

Widely used SRT can be broadly categorized into two groups: (1) imaging-based approaches, and (2) next-generation sequencing (NGS)-based approaches [6–10]. These diverse technologies simultaneously capture both gene expression and spatial location, while differing in gene throughput, sensitivity, resolution, and field of view. Imaging-based methods provide subcellular resolution with high sensitivity but limited gene throughput [11–17], while NGS-based technologies generate diverse spatial resolutions, ranging from multicellular to subcellular levels, for the captured spots with whole-transcriptome coverage but lower sensitivity [18–20].

Several computational methods have been developed to identify spatial domains in SRT data. Previous studies utilized traditional methods (*e*.*g*., K-means [21,22] and Louvain [23]), which did not take into account the spatial information of SRT data, leading to a discontinuous division of regions. Recent methods have introduced spatial information to improve spatial domain identification. For example, BayesSpace [24] employed a Bayesian statistical approach, using the information from spatial neighborhoods for spatial clustering. Deep learning methods have also been developed to integrate spatial information and gene expression of SRT data. StLearn [25] defined morphological distances based on features extracted from histology images and utilized these distances, along with spatial neighbors, to smooth gene expression. SpaGCN [26] also used histology images by employing graph convolutional networks to integrate gene expression, spatial location, and image data. SpaceFlow [27] utilized spatially regularized deep graph networks to integrate both expression similarity and spatial information. STAGATE [28] applied a graph attention auto-encoder to learn low-dimensional latent embeddings of SRT profiles. Although these methods have shown significant improvements on some datasets, they primarily focus on the cell-cell pairwise relationships, neglecting the higher-order structures inherent in multicellular programs. Furthermore, they fail to account for the delineation of domain boundaries, a crucial aspect for improving spatial domain identification, as they predominantly focus on clustering. This limitation becomes more pronounced when confronted with higher spatial resolutions, making it challenging to elucidate the tissue structure comprehensively. Moreover, these approaches encounter difficulties in extending their capabilities to accommodate other omics.

To tackle these issues, we propose HyperSTAR, the first hypergraph learning-based spatial clustering method that explicitly learns the higher-order tissue structures. HyperSTAR can effectively leverage complex relationships among spatially adjacent multicellular programs. A gene expression-guided hyperedge decomposition module and a hypergraph attention convolutional layer are employed to precisely delineate boundaries of spatial domains. Extensive experiments show that HyperSTAR can reveal tissue structure and tumor spatial heterogeneity across varying spatial resolutions, demonstrating effectiveness in diverse downstream analyses, such as spatiotemporal trajectory inference, gene expression denoising, and spatially variable gene (SVG) detection.

## Method

### Data description

We applied HyperSTAR to a diverse range of spatial omics datasets generated by various technologies to demonstrate its effectiveness. These datasets span multiple platforms, including SRT technologies such as 10x Visium, Stereo-seq, ouroboros single-molecule fluorescence *in situ* hybridization (osmFISH), multiplexed error-robust fluorescence *in situ* hybridization (MERFISH), and 10x Xenium, as well as CODEX for spatial proteomics. These platforms produce data with varying resolutions, ranging from spot-level to single-cell resolution. For example, 10x Visium datasets have a resolution of 55 µm (1–10 cells per spot), while Stereo-seq provides spot resolutions with bin sizes of 20, 50, and 100 µm, as well as single-cell resolution.

Specifically, we used the human dorsolateral prefrontal cortex (DLPFC) dataset [29], generated by 10x Visium, as the benchmark for spatial domain identification. This dataset includes 12 human DLPFC sections from 3 individuals, with manual annotations provided by the original authors. The 10x Visium mouse brain coronal dataset comprises 2702 spots and 21,949 genes as raw features. The Stereo-seq mouse brain olfactory bulb dataset, with single-cell resolution, includes 19,109 spots and 27,106 genes as raw features. The breast cancer section dataset, generated by 10x Visium, has a median of 20,762 unique molecular identifiers (UMIs) per spot and a median of 6026 genes per spot. These datasets cover diverse species, organs, and resolutions, showing the versatility of HyperSTAR.

### Data preprocessing

To effectively analyze the raw data, we employed a series of preprocessing steps. First, we removed spots located outside the primary tissue region to focus on biologically relevant areas. Next, we performed feature selection to identify the top highly variable genes (HVGs) (typically 3000 genes). After selecting the HVGs, we applied a log transformation to the gene expression values. The resulting normalized expression values of HVGs were then used as input node representations in the hypergraph learning module. This preprocessing ensures that the hypergraph learning module can effectively capture the intricate relationships among spots and identify biologically meaningful spatial domains.

### Hypergraph construction

After preprocessing to obtain spot representations, we constructed the hypergraph structure in two steps:

Step 1. Initial hyperedge construction: For each spot, we generated a hyperedge using the k-nearest neighbors (kNN) approach based on spatial information of the spots. This step captures local spatial relationships among spots, ensuring that spatially adjacent spots are connected in the hypergraph.

Step 2. Hyperedge refinement: To address the scenarios where spatially adjacent spots belong to different biological domains, we refined the hyperedge set using pre-clustering results based on gene expression. This refinement aligns the hypergraph structure with underlying biological domains, thereby improving the accuracy of spatial domain identification. Specifically, if the spots within a hyperedge belong to the same cluster, the hyperedge remains unchanged, indicating that spatially adjacent spots share similar gene expression patterns and likely belong to the same biological domain. If the spots within a hyperedge belong to different clusters, we consider two scenarios: (1) Single outlier spot: If only one spot belongs to a different cluster, it is removed from the hyperedge to maintain consistency. This ensures that the hyperedge only contains spots that are both spatially adjacent and share similar gene expression patterns; (2) Multiple outlier spots: If multiple spots belong to different clusters, the hyperedge is decomposed into two new hyperedges, each containing spots from the same cluster. This effectively separates the spots that are spatially adjacent but have distinct gene expression patterns. By refining the hyperedge set based on the pre-clustering results, we better capture the spatial and biological relationships among spots, leading to a more accurate representation of spatial domains in the hypergraph structure.

### Hypergraph learning

Once the hypergraph is constructed, we input the spot features and hypergraph structure into the hypergraph learning module, which consists of two parts: the encoder and the decoder. The encoder generates low-dimensional latent embeddings of spots, used for spatial domain identification and downstream analyses. The decoder produces reconstructed features of spots, utilized for denoising.

Given a hypergraph **G** = (**X**, **H**), where $X\in \;R^{n\times d}$ represents the feature matrix of *n* spots and $H\in \;R^{n\times m}$ represents the incidence matrix from vertexes to hyperedges defined as [30,31]:


(1) $$h_{ij}=\{\begin{array}{cc} 1, & \;\text{if}\;x_{i}\in N_{j}^{e}\\ 0, & \;\text{otherwise} \end{array}$$

where $N_{j}^{e}$ denotes the set of vertexes connected to the *j*-th hyperedge.

#### Encoder

The encoder takes the normalized gene expression $X_{0}$ as input and generates the spot embeddings by collectively aggregating the information from its neighbors in the same hyperedge. To adaptively learn the weights of the hyperedges, we use three hypergraph attention layers.

#### Hypergraph convolutional layer

Message propagation on a hypergraph is more complex than that on a graph, involving two stages: attentive hyperedge aggregation and attentive vertex aggregation [32]. In the attentive hyperedge aggregation, the connected vertexes in the same hyperedge are aggregated to generate the hyperedge embeddings. As for the *t*-th layer, the hyperedge features $E_{t-1}$ can be calculated as:


(2) $$E_{t-1}=H^{T}X_{t-1}$$

where $X_{t-1}$ represents the output vertex features of the previous layer. For each pair of hyperedges ($e_{i},e_{j}$) where $e_{j}\in \;N_{i}^{v}$, we calculate the coefficient $a_{ij}$ between hyperedge i and hyperedge j by introducing the attention mechanism [33]:


(3) $$a_{ij}=\frac{\text{exp}(\text{LeakyReLU}({\vec{\theta }}_{a}\left[W_{a}\vec{e_{i}}∥W_{a}\vec{e_{j}}\right]))}{\sum_{e_{k}\in N_{i}^{v}}\;\text{exp}(\text{LeakyReLU}({\vec{\theta }}_{a}\left[W_{a}\vec{e_{i}}∥W_{a}\vec{e_{k}}\right]))}$$

where $W\in \;R^{d\prime \times d}$ is a shared linear transformation, parametrized by a weight matrix. ${\vec{\theta }}_{a}\in \;R^{2d\prime }$ represents a shared attentional mechanism, parametrized by a vector. $N_{i}^{v}$ denotes the set of hyperedges connected to the *i*-th vertex. The hyperedge features aggregated by attentive hyperedge aggregation can be calculated as follows:


(4) $$E_{t}=\mathrm{AW}H^{T}X_{t-1}$$

The attentive vertex aggregation module aggregates the connected hyperedge information to generate the vertex embeddings. First, the vertex features can be calculated by


(5) $$X_{t}^{e}=HE_{t}$$

We calculate the coefficient $b_{ij}$ between hyperedge i and vertex j based on the attention mechanism:


(6) $$b_{ij}=\frac{\text{exp}(\text{LeakyReLU}({\vec{\theta }}_{b}\left[\vec{x_{i}^{e}}∥\vec{x_{j}^{e}}\right]))}{\sum_{x_{k}\in N_{i}^{e}}\;\text{exp}(\text{LeakyReLU}({\vec{\theta }}_{b}\left[\vec{x_{i}^{e}}∥\vec{x_{k}^{e}}\right]))}$$

where ${\vec{\theta }}_{b}\in \;R^{2d\prime }$ represents a shared attentional mechanism, parametrized by a vector, while $N_{i}^{e}$ denotes the set of vertexes connected to the *i*-th hyperedge. Next, the output vertex features $X_{e}$ can be calculated by


(7) $$X_{e}=\sigma (\mathrm{BHAW}H^{T}X_{t-1})$$

where σ is the activation function.

#### Decoder

The decoder takes the low-dimensional latent embeddings of spots as input and reconstructs the spot features in the original input dimensions of the encoder. We also use hypergraph attention networks as input to build a symmetrical decoder for the encoder. After applying three layers of hypergraph attention networks, the output of the last layer is considered as the reconstructed spot features. The output of the *t*-th layer in the decoder can be obtained by:


(8) $${\hat{X}}_{t}=\sigma ({\hat{X}}_{t-1}HW_{k-t+1}^{T}A_{k-t+1}^{T}H^{T}B_{k-t+1}^{T})$$

where ${\hat{X}}_{0}\;$ is the latent embeddings learned by the encoder, while *k* represents the number of layers of the encoder or decoder in our model. We set *k* = 3 in this study.

#### Loss function

Hypergraph-structured data include node features and the graph structure, both of which should be encoded by high-quality node representations. We minimize the reconstruction loss of node features as follows:


(9) $$\sum_{i=1}^{N}∥X_{in}-X_{\mathrm{out}}{∥}_{2}$$

where $X_{in}$ is the input normalized gene expression $X_{0}$ of the encoder, and $X_{\mathrm{out}}={\hat{X}}_{k}$ represents the final output of the decoder.

### Downstream analysis

#### Clustering

To identify spatial domains based on the embeddings obtained by HyperSTAR, we employed different strategies depending on the availability of prior information about the number of labels or domains. When the number of labels is known, we utilized the mclust clustering algorithm [34]. For data without prior information about the number of domains, we used the Louvain algorithm implemented in the Scanpy package. By employing these two different clustering strategies based on the availability of prior information, we can flexibly adapt the spatial domain identification process to various datasets and research questions.

#### Spatial trajectory inference

To visualize spatial trajectories, we employed two complementary approaches: the Uniform Manifold Approximation and Projection (UMAP) plot and the partition-based graph abstraction (PAGA) graph [35]. The UMAP plot is used to visualize the embeddings generated by HyperSTAR. The PAGA graph is another powerful tool for visualizing spatial trajectories. PAGA is a graph-based method that combines clustering with trajectory inference, allowing for the identification of both discrete cell states and continuous transitions between them. In the context of spatial omics data, the PAGA graph can reveal the relationships between spatial domains and potential trajectories connecting them. We generated the PAGA graph using the scanpy.pl.paga function from the Scanpy package, which takes the HyperSTAR embeddings as input and produces a visually interpretable graph representation of the spatial trajectories. By combining the UMAP plot and the PAGA graph, we can gain complementary and valuable insights into the spatial organization and potential trajectories present in the data, thereby facilitating the interpretation of the underlying biological processes.

#### SVG detection

We performed the Wilcoxon test implemented in the Scanpy package to identify SVGs for spatial domains with a Benjamini–Hochberg-corrected false discovery rate (FDR) threshold of 1%.

#### Denoising

The denoised gene expression is generated by the decoder, and the visualization demonstrates that the denoised features are more effective and representative for distinguishing different domains.

## Results

### Overview of HyperSTAR

**Figure 1:**
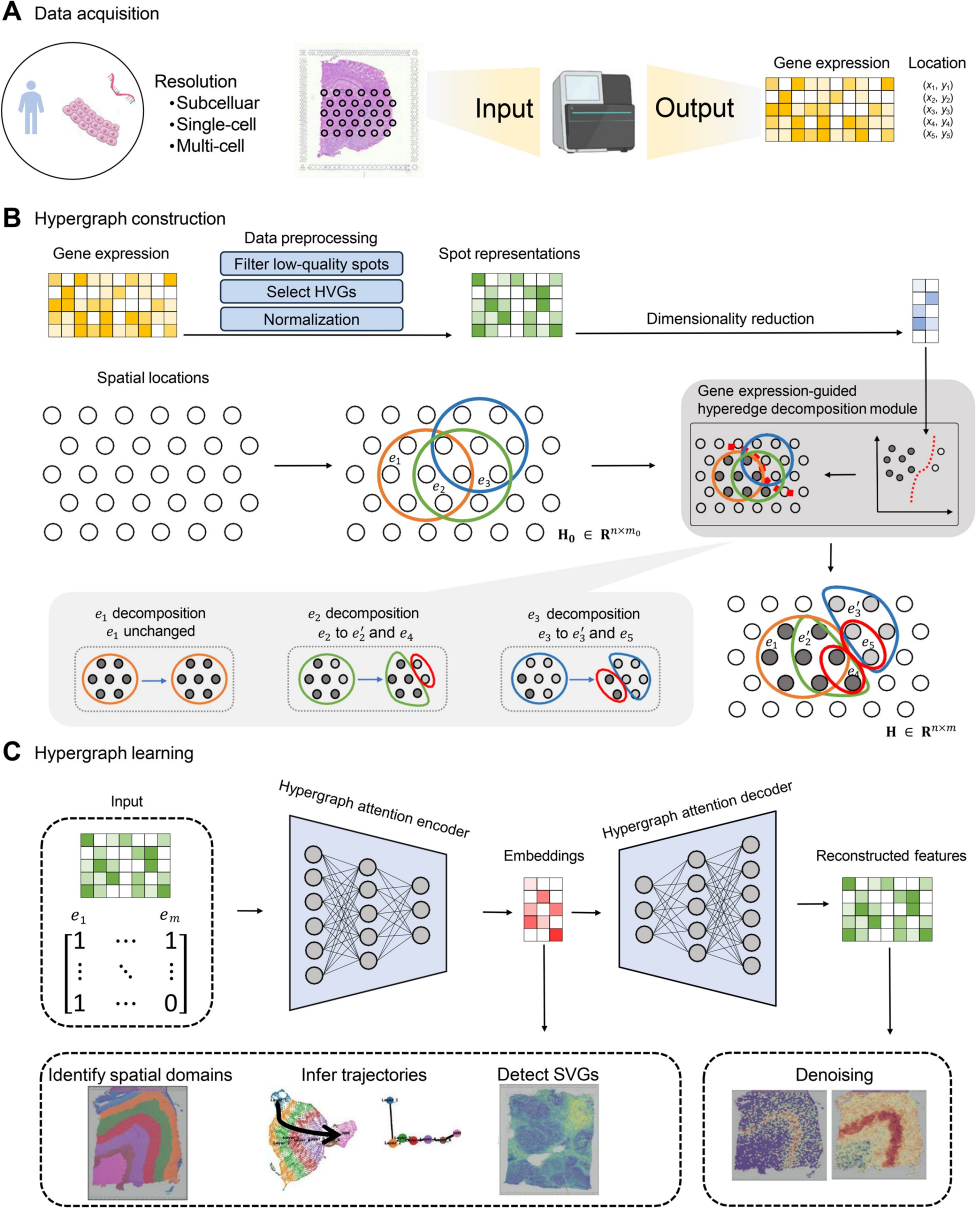
Overview of HyperSTAR **A**. SRT data vary in resolution depending on the sequencing technology. Typically, SRT data consist of gene expression profiles and their corresponding spatial coordinates. **B**. A hypergraph is constructed to model relationships among multi-spots. Spot representations are derived directly from gene expression data. The hypergraph structure is built in two stages: (1) for each spot, a hyperedge is formed by connecting its spatially adjacent multi-spots, and (2) hyperedge decomposition is performed to refine the hyperedges based on gene expression clustering results. **C**. The refined hyperedge set and spot representations are input into hypergraph attention convolutional neural networks to learn latent embeddings of spots for downstream analyses. SRT, spatially resolved transcriptomics; HVG, highly variable gene; SVG, spatially variable gene.

SRT data typically contain a gene expression matrix and a spatial coordinate matrix, as illustrated in **Figure 1**A. To comprehensively capture the intricate associations among spatially adjacent multiple spots, we develop a hypergraph-based method, as illustrated in Figure 1B and C. The construction of this hypergraph can be primarily divided into two key steps: (1) acquiring spot representations and (2) establishing the hypergraph structure. Spot representations are acquired from preprocessed gene expression data. The hypergraph structure is attained through two stages: (1) constructing a naive hyperedge set among spatially neighboring multi-spots, and (2) employing a gene expression-guided hyperedge decomposition module to refine these naive hyperedges. Finally, a hypergraph structure that integrates spatial information and gene expression is obtained (Figure 1B). The initially built naive hyperedge set contains complex high-order relationships among spatially neighboring multi-spots. However, it is important to note that adjacent spots in space do not always belong to the same domain. For instance, although spots near a domain boundary are spatially adjacent, they might actually belong to different domains. To tackle this issue, a gene expression-guided hyperedge decomposition module is employed to enhance the effects of gene expression. After constructing the hypergraph, we utilize hypergraph attention convolutional neural networks to learn low-dimensional latent embeddings of the SRT data. Subsequently, these latent embeddings are used to visualize data, identify spatial domains, infer trajectories, detect SVGs, and denoise spatial gene expression (Figure 1C). Using the aforementioned strategy to integrate spatial information and gene expression in SRT data, HyperSTAR improves the performance of spatial omics analysis.

### HyperSTAR achieves state-of-the-art performance on the benchmark dataset

**Figure 2:**
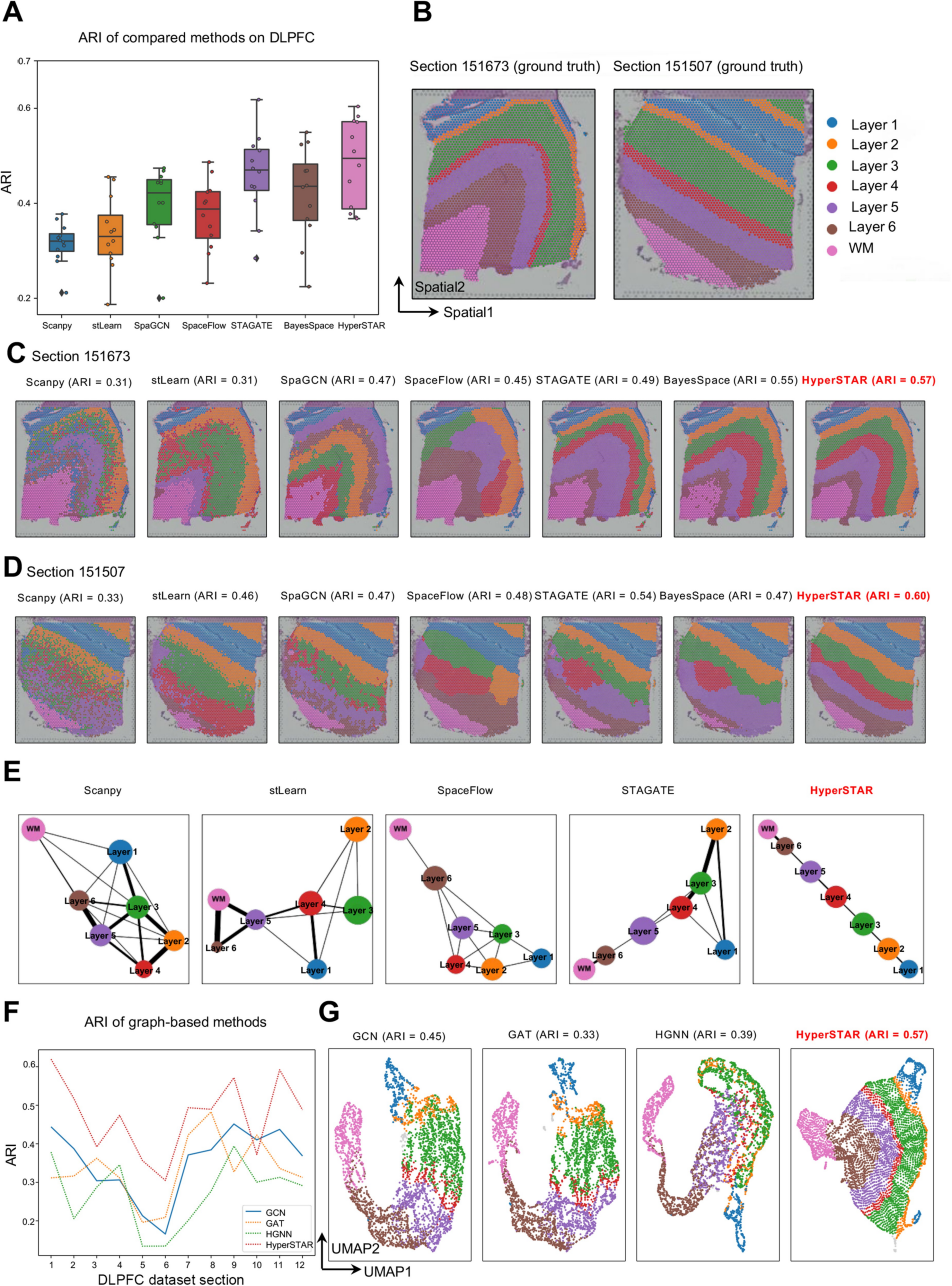
HyperSTAR achieves state-of-the-art performance on the DLPFC benchmark dataset **A**. Box plots of clustering accuracy across all 12 sections of the DLPFC dataset, measured by ARI scores for 7 methods. **B**. Ground-truth segmentation of cortical layers (from Layer 1 to Layer 6) and WM in sections 151673 and 151507. **C**. Spatial visualization of clustering results of HyperSTAR in section 151673 compared to six state-of-the-art methods. **D**. Spatial visualization of clustering results of HyperSTAR in section 151507 compared to six state-of-the-art methods. **E**. PAGA graphs generated by Scanpy, stLearn, SpaceFlow, STAGATE, and HyperSTAR. **F**. Line plot of clustering accuracy across all 12 sections of the dataset, measured by ARI scores for four different graph-based networks (GCN, GAT, HGNN, and HyperSTAR). **G**. UMAP plots of section 151673 obtained by the four graph-based networks. DLPFC, dorsolateral prefrontal cortex; WM, white matter; UMAP, Uniform Manifold Approximation and Projection; ARI, Adjusted Rand Index; PAGA, partition-based graph abstraction; GCN, graph convolution network; GAT, graph attention network; HGNN, hypergraph convolution network.

To evaluate the performance of HyperSTAR, we first used the widely recognized SpatialLIBD dataset, a 10x Visium dataset of 12 human DLPFC sections [29]. This dataset includes manually annotated DLPFC layers (Layer 1 to Layer 6) and white matter (WM), which are defined based on morphological features and marker genes. Using these annotations as the ground truth, we employed multiple established metrics — Adjusted Rand Index (ARI), Normalized Mutual Information (NMI), Moran’I, and Geary’s C — to quantitatively assess the accuracy and robustness of spatial domain identification (**Figure 2**A, Figure S1).

We compared HyperSTAR with six commonly used packages for identifying spatial domains, including Scanpy [23] (originally designed for single-cell omics), two methods that integrate histology images (stLearn and SpaGCN), two deep learning methods based on graphs (SpaceFlow and STAGATE), and one Bayesian statistical method (BayesSpace). HyperSTAR effectively identified the expected cortical layer structures, demonstrating significant improvement compared to other methods (Figure 2A). For example, in sections 151673 and 151507 (ground truth shown in Figure 2B), HyperSTAR clearly delineated the layer boundaries and achieved the highest clustering accuracy (ARI = 0.57 and ARI = 0.60, respectively) (Figure 2C and D). The results of spatial visualization for all sections are provided in Figures S2–S16. We further confirmed the inferred trajectory using a trajectory inference algorithm named PAGA. The PAGA graphs illustrate that embeddings belonging to different clusters obtained by Scanpy, stLearn, and SpaceFlow are intermingled. As for STAGATE, it successfully separates WM, Layer 6, and Layer 5; however, Layer 1, Layer 2, Layer 3, and Layer 4 remain mixed. In contrast, our HyperSTAR embeddings effectively separate these cortical layers, providing a clear depiction of tissue topology (Figure 2E).

We further conducted ablation experiments to illustrate the effectiveness of our strategies. As shown in Figure 2F, we evaluated the performance of four graph-based networks: graph convolution network (GCN) [36], graph attention network (GAT) [33], hypergraph convolution network (HGNN) [30], and our HyperSTAR, which combines hypergraph convolution operation and a graph attention mechanism. The results demonstrate that HyperSTAR achieves higher ARI scores than other methods on almost all sections of the DLPFC dataset (Figure 2F). As shown in Figure 2G, UMAP plots of embeddings obtained from the four networks demonstrate the superiority of HyperSTAR. For GCN, GAT, and HGNN, there is some overlap between cortical layers, indicating that some spots are mixed. In contrast, HyperSTAR demonstrates exceptional performance by clearly distinguishing all cortical layers without any overlap or mixing. To ensure the robustness of our findings, we included multiple running results in Figure S17. Additionally, the denoising results and the detection of SVGs are presented in Figures S18 and S19, respectively.

### HyperSTAR reveals a substructure at finer resolutions in the mouse olfactory bulb

**Figure 3:**
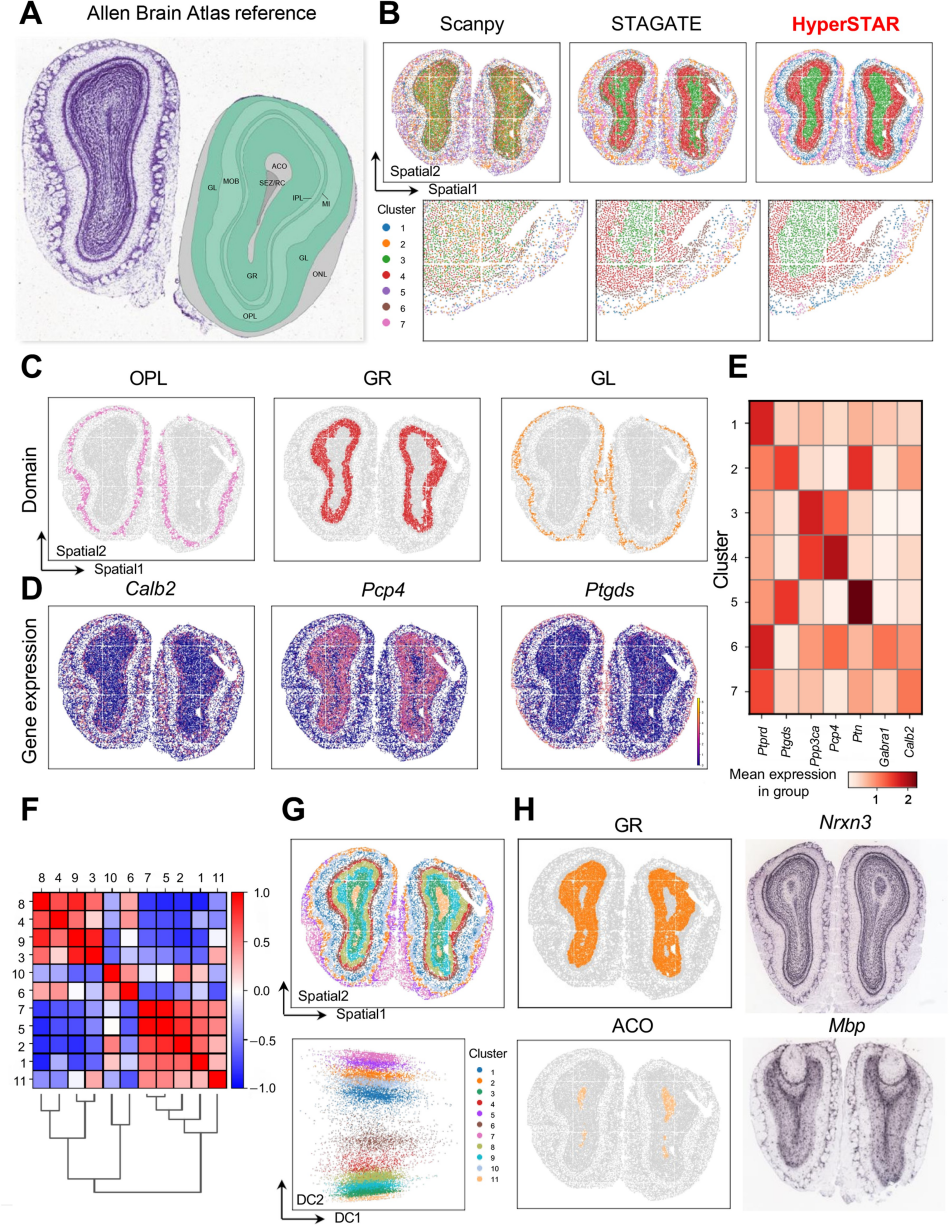
HyperSTAR reveals the ACO substructure at finer resolutions in the Stereo-seq MOB **A**. MOB anatomical structure from the Allen Brain Atlas. **B**. Spatial visualization of results obtained by Scanpy, STAGATE, and HyperSTAR. **C**. The OPL, GL, and GR layers identified by HyperSTAR. **D**. Spatial visualization of three marker genes expressed in OPL (*Calb2*), GL (*Pcp4*), and GR (*Ptgds*), respectively. **E**. Heatmap displaying top genes characterizing seven groups identified by HyperSTAR. **F**. Correlation matrix of HyperSTAR results, showing similarity within clusters and dissimilarity between clusters. **G**. Top: spatial visualization of results from HyperSTAR. Bottom: diffusion map revealing the ACO substructure within GR. **H**. Top: GR identified by HyperSTAR and ISH image of its marker gene *Nrxn3* from the Allen Brain Atlas; Bottom: ACO identified by HyperSTAR and ISH image of its marker gene *Mbp* from the Allen Brain Atlas. MOB, mouse olfactory bulb; SEZ/RC, subependymal zone; ACO, anterior commissure; GR, granular layer; IPL, inner plexiform layer; MI, mitral layer; OPL, outer plexiform layer; GL, glomerular layer; ONL, olfactory nerve layer; DC, dorsal cochlear; ISH, *in situ* hybridization.

To assess HyperSTAR’s performance across different resolutions, we selected a mouse olfactory bulb (MOB) section from Stereo-seq, with finer spatial resolution at the single-cell level. Spatial domains on this section were initially identified using three methods: Scanpy, a widely used single-cell method; STAGATE, a graph-based deep learning method; and our proposed method, HyperSTAR. Using mouse anatomy collected from the Allen Brain Atlas as reference (**Figure 3**A), Scanpy yielded discontinuous results, while STAGATE showed improved performance, albeit with some mixing between layers, such as the fusion of domain 3 and domain 4 (Figure 3B). In contrast, HyperSTAR identified a clear and continuous layer structure in the MOB.

To further analyze the results obtained by HyperSTAR, we ranked genes for characterizing groups, and the top genes identified for each group are shown in Figure 3E. The gene expression of *Calb2*, *Pcp4*, and *Ptgds*, as shown in Figure 3D, represents the three top genes identified by HyperSTAR for characterizing domains 7, 4, and 2, respectively. The distribution of domains 7, 4, and 2 identified by HyperSTAR is displayed in Figure 3C, corresponding to the outer plexiform layer (OPL), granular layer (GR), and glomerular layer (GL) in the reference shown in Figure 3A. The spatial visualization and corresponding *in situ* hybridization (ISH) images of marker genes (distinguishing different layers) from the Allen Brain Atlas are also provided in Figures S20 and S21. These results collectively demonstrate HyperSTAR’s ability to reveal continuous tissue structure and identify effective marker genes.

We adjusted the parameters for fine-grained clustering, and the result is shown in Figure 3G. HyperSTAR reveals a substructure, anterior commissure (ACO), within GR, together with its top mark gene *Mbp*  (Figure 3H). Additionally, we identified *Nrxn3* as the top mark gene for GR. The ISH images illustrate the effectiveness of the results obtained by HyperSTAR. The heatmap of the correlation matrix of clustering also demonstrates the effectiveness of HyperSTAR, showing similarity within clusters and dissimilarity between clusters (Figure 3F).

We further assessed HyperSTAR’s cross-resolution capability on the aforementioned MOB section at various spatial resolutions. Across bin 20, bin 50, bin 100, and single-cell levels (Figures S22 and S23), our HyperSTAR results consistently exhibited a continuous structure aligned with the reference shown in Figure 3A. Extending our evaluation to an adult axolotl brain slice further confirmed HyperSTAR’s efficacy, revealing a continuous structure corresponding to the reference (Figure S24).

### HyperSTAR unveils spatial intra-tumor heterogeneity in the breast cancer section and its association with survival

**Figure 4:**
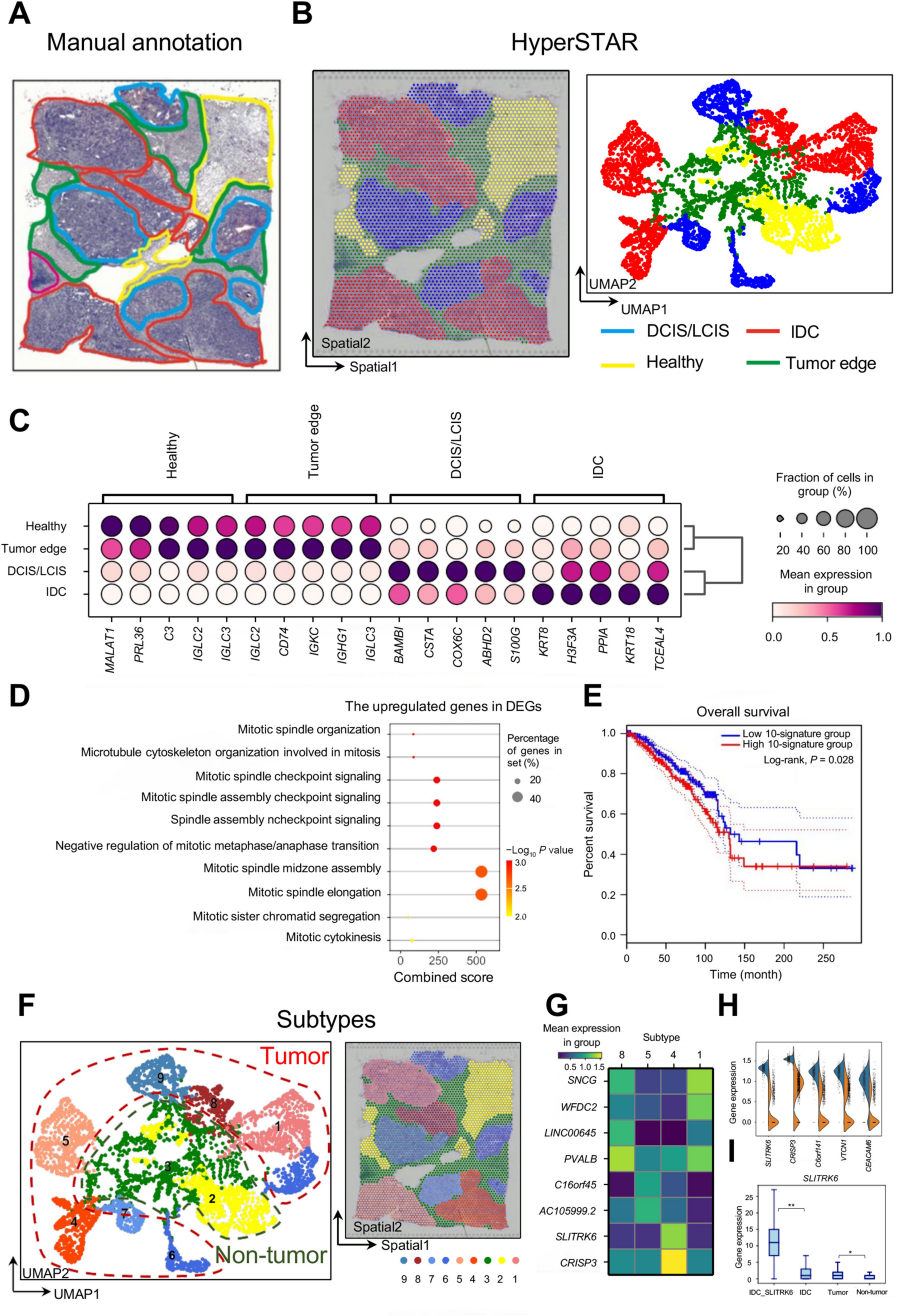
HyperSTAR identifies marker genes in breast cancer subtypes and their association with survival **A**. Manual annotation of a breast cancer section from 10x Visium by pathology experts. **B**. Spatial result and UMAP plot generated by HyperSTAR for this section. **C**. Dot plot of marker genes identified by HyperSTAR. **D**. Gene Ontology enrichment analysis of significantly upregulated genes in IDC compared to the rest of the section. **E**. Overall survival curves for patients with 10 signature genes for IDC, analyzed using TCGA breast cancer gene expression data via GEPIA2. **F**. Subtypes identified by HyperSTAR in the breast cancer section. **G**. Heatmap showing the expression of marker genes in IDC subtypes. **H**. Expression levels of the top 5 marker genes of the IDC_SLITRK6 subtype compared to the rest of the IDC subtypes. **I**. Expression levels of *SLITRK6* in IDC_SLITRK6, IDC, tumor, and non-tumor regions. Significant difference was determined by Wilcoxon rank-sum test (IDC_SLITRK6 *vs*. IDC: **, *P* < 0.01; Tumor *vs*. Non-tumor: **, *P* < 0.05). DCIS/LCIS, ductal carcinoma *in situ*/lobular carcinoma *in situ;* IDC, invasive ductal carcinoma; DEG, differentially expressed gene; TCGA, The Cancer Genome Atlas.

To investigate the capability of HyperSTAR for tumor analysis, we applied it to a breast cancer section obtained from 10x Visium. This section was manually annotated by a pathologist into four distinct regions: ductal carcinoma *in situ*/lobular carcinoma *in situ* (DCIS/LCIS), invasive ductal carcinoma (IDC), healthy tissue, and the tumor edge (**Figure 4**A) [37]. Remarkably, HyperSTAR accurately identified these regions, demonstrating high consistency with the manual annotations (Figure 4B, Figure S25).

We next interrogated the diagnostic power of HyperSTAR’s results. We ranked and plotted the top 5 genes for characterizing these 4 domains (Figure 4C). *KRT8* and *KRT18* were highly expressed in the IDC region. *KRT8* is an independent prognostic indicator for poor survival in lung adenocarcinoma [38]. *KRT18* often acts together with *KRT8* in interleukin-6 (IL-6)-mediated barrier protection. This patient has been clinically diagnosed as Grade III. Walker et al. claimed that *KRT8/18* expression differentiates distinct subtypes of Grade III IDC of the breast cancer [39], which is consistent with our results. We further compared transcriptional differences between IDC and the remaining regions by performing differential expression analysis followed by Gene Ontology enrichment analysis. The significantly upregulated genes in IDC were enriched in mitotic processes, indicating abnormal proliferation of cancer cells (Figure 4D). This suggests that the IDC region is probably infiltrated with cancer cells and highly active. We further investigated the correlation of these significantly upregulated genes with survival using an independent clinical dataset from The Cancer Genome Atlas (TCGA) breast cancer cohort. Ten signature genes were selected to stratify patients. The result showed that low expression of these ten signature genes correlated with shorter overall survival (Figure 4E). Hence, these results indicate a possible developmental trajectory from cancer stem cells to malignant cells. Furthermore, some studies have indicated that *KRT8* is a pan-cancer indicator [40]. In light of this, we conducted pan-cancer survival analysis using the significantly upregulated genes identified in IDC. The outcomes further revealed a correlation between these significantly upregulated genes and pan-cancer development (Figure S26).

We further investigated the subtypes within the IDC region. According to our HyperSTAR result, the IDC region is divided into four subtypes (Figure 4F). The heatmap, generated by the top 2 genes of each subtype (Figure 4G), highlights the unique expression profiles within each subtype. Notably, for domain 4, *SLITRK6* and *CRISP3* exhibited high expression levels, with *SLITRK6* emerging as a potential marker for this specific subtype (Figure 4H). The accompanying box plot showed that *SLITRK6* was significantly upregulated in IDC_SLITRK6 compared to IDC, tumor, and non-tumor regions (Figure 4I). In summary, HyperSTAR not only enables the identification of intra-tumor heterogeneity but also facilitates the exploration of distinct subtypes within the intra-tumoral environment.

### HyperSTAR is generalizable

**Figure 5:**
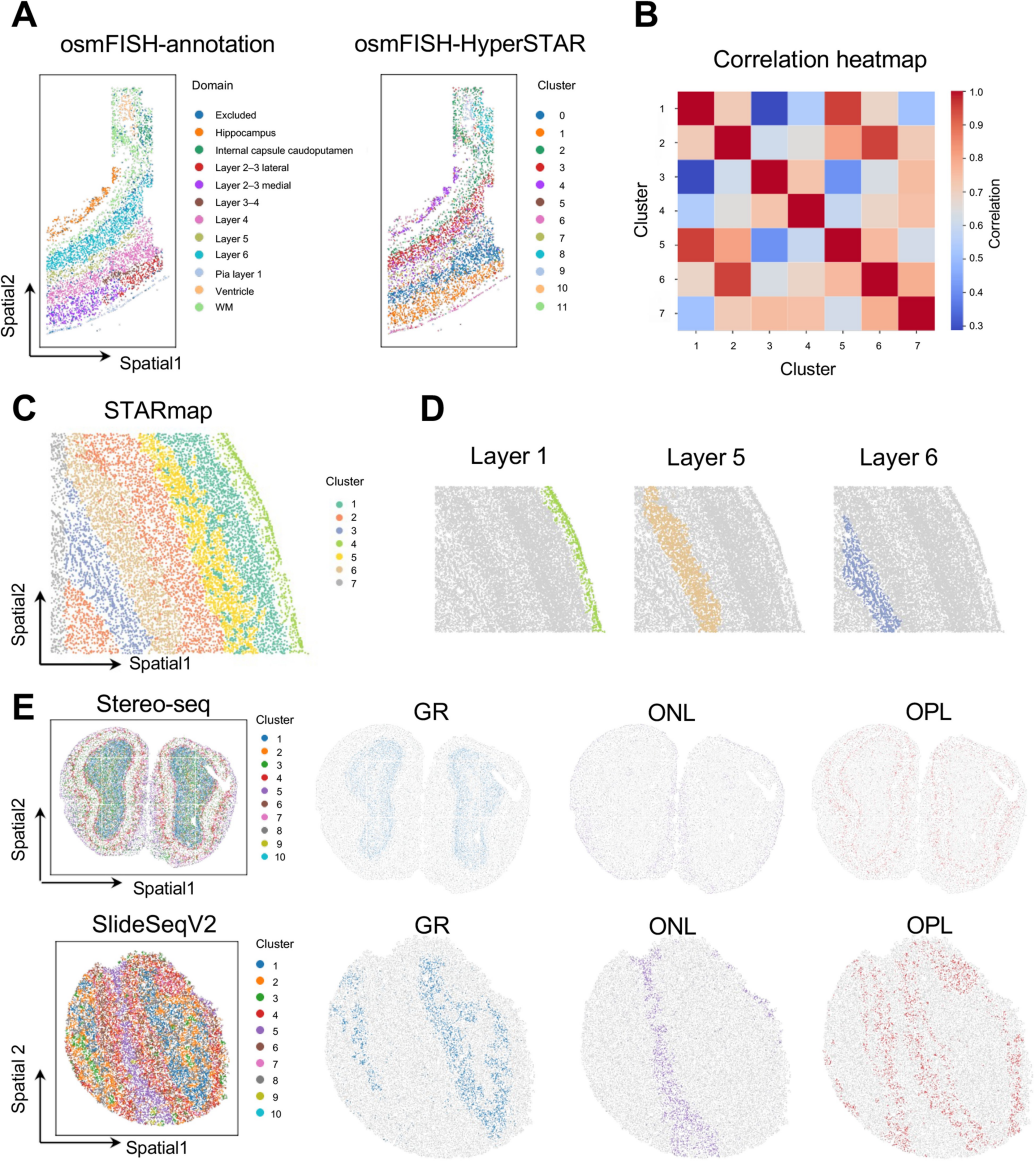
HyperSTAR is applicable to various spatial omics datasets and demonstrates joint clustering capabilities **A**. Manual annotation and HyperSTAR-based identification of the somatosensory cortex from osmFISH data. **B**. Correlation heatmap of clustering results obtained by HyperSTAR on STARmap data. **C**. Spatial results of HyperSTAR on STARmap data from a mouse brain section. **D**. Spatial distribution of different layers revealed by HyperSTAR on STARmap data. **E**. Joint clustering of Stereo-seq and SlideSeqV2 data by HyperSTAR. The top panel shows results for Stereo-seq data: clustering results (left) and spatial distribution of different layers identified by HyperSTAR (right). The bottom panel shows results for SlideSeqV2 data: clustering results (left) and spatial distribution of different layers identified by HyperSTAR (right). osmFISH, ouroboros single-molecule fluorescence *in situ* hybridization.

To comprehensively validate the versatility of HyperSTAR across various technologies and different omics, we applied it to multiple platforms, including 10x Genomics (10x Visium and 10x Xenium), MERFISH [41], NanoString (CosMx), and osmFISH (**Figure 5**, Figure S27). Our evaluation was further extended to different omics layers, encompassing both transcriptomics and proteomics, demonstrating the robust adaptability and broad applicability of HyperSTAR.

HyperSTAR reveals the cortex distribution of the somatosensory cortex (Figure 5A) in a dataset produced by osmFISH, a technique based on multiplexed fluorescence *in situ* hybridization [42]. osmFISH enables the detection of hundreds of genes at single-cell resolution, providing valuable insights into the spatial organization of complex tissues. By applying HyperSTAR to this dataset, we identified distinct layers and regions within the somatosensory cortex, showcasing HyperSTAR’s ability to uncover biologically meaningful structures. To further demonstrate the versatility of HyperSTAR, we applied it to the STARmap mouse brain data [43]. The resulting correlation heatmap of the clustering results showed high correlations within clusters but low correlations between clusters (Figure 5B). This indicates that HyperSTAR successfully captures the underlying structure of the STARmap data, grouping domains with similar spatial gene expression profiles while separating domains from different layers or regions. HyperSTAR explicitly revealed the distribution of brain layers (Figure 5C and D), highlighting its effectiveness in identifying spatial patterns across different experimental platforms.

The hypergraph framework is a highly scalable and versatile approach that can be applied to jointly cluster data from multiple sources. To evaluate its capability, we selected MOB SRT data from two different sequencing platforms: SlideSeqV2 and Stereo-seq. These datasets exhibited differences in quality and distribution (Figure S28). To integrate data from these platforms, we extended the hypergraph construction process beyond single slices. In addition to creating hyperedges within each slice, we established inter-slice relationships by constructing hyperedges that connect spots across slices based on their normalized log-transformed gene expression profiles. This approach allows for the identification of shared spatial patterns across different datasets.

To address potential batch effects arising from different sequencing platforms, we utilized Harmony for batch effect correction. The joint clustering results generated by HyperSTAR (Figure 5E, Figure S29) successfully delineated distinct layers within the MOB, aligning with its known anatomical structure. By leveraging the complementary information provided by SlideSeqV2 and Stereo-seq, HyperSTAR effectively identified shared spatial patterns across datasets, offering a more comprehensive understanding of tissue organization.

## Discussion

The advent of spatial omics technologies has revolutionized our understanding of biological processes by enabling the simultaneous acquisition of biomolecular and spatial information. Accurate identification of spatial domains is crucial for deciphering spatiotemporal life processes. In this study, we introduce HyperSTAR, a powerful and versatile method for spatial omics analysis that excels in accurately identifying spatial domains, even in high-resolution spatial data. By incorporating a gene expression-guided hyperedge decomposition module and a hypergraph attention convolutional neural network, HyperSTAR demonstrates exceptional precision in recognizing spatial domains across varying resolutions. The advancements hold significant implications for exploring organ development and tumor microenvironments.

Benchmarking and comparisons with existing advanced graph neural network models underscore HyperSTAR’s superior performance in uncovering tissue substructures, inferring spatiotemporal patterns, and denoising spatially resolved gene expression. Its application to spatial omics data from diverse platforms — including 10x Genomics, NanoString, CODEX, and osmFISH — highlights its adaptability and robustness across different technologies. Furthermore, the validation of HyperSTAR’s findings through functional and survival analyses of independent clinical data, particularly in the context of breast cancer, reinforces its utility in deciphering spatial heterogeneity and its potential for clinical relevance.

A key strength of HyperSTAR lies in its ability to capture intricate higher-order relationships among spatially neighboring multi-spots. However, the current version of HyperSTAR is primarily focused on identifying spatial domains and has yet to fully exploit the potential of hypergraphs. In future work, we aim to extend HyperSTAR to integrate multi-omics data from spatial omics experiments generated by different platform technologies. Additionally, incorporating histology images could provide valuable insights for addressing clinically relevant questions and further enhancing the method’s applicability.

In conclusion, HyperSTAR addressed challenges associated with diverse spatial resolutions and enabling the exploration of complex biological systems. Its ability to capture intricate higher-order relationships within spatially neighboring multi-spots makes it an invaluable tool for researchers studying organ development, tumor microenvironments, and other spatially resolved biological processes. This study establishes HyperSTAR as a versatile and effective computational approach with broad applicability across diverse spatial omics datasets and large-scale analyses. Future directions include refining and expanding HyperSTAR’s capabilities, integrating multi-omics data and histology images, and exploring its application in additional biological contexts.

## Code availability

The HyperSTAR method is implemented in Python. It is available on GitHub (https://github.com/Ringoio/HyperSTAR). The code has also been submitted to BioCode at the National Genomics Data Center (NGDC), China National Center for Bioinformation (CNCB) (BioCode: BT007916), which is publicly accessible at https://ngdc.cncb.ac.cn/biocode/tools/BT007916.

## CRediT author statement

**Yi Liao:** Conceptualization, Methodology, Investigation, Data curation, Formal analysis, Writing – original draft, Writing – review & editing. **Chong Zhang:** Investigation, Data curation, Formal analysis, Writing – review & editing. **Zhikang Wang:** Conceptualization, Methodology, Visualization, Writing – review & editing. **Fei Qi:** Conceptualization, Methodology, Writing – review & editing. **Weitian Huang:** Conceptualization, Methodology, Writing – review & editing. **Shangyan Cai:** Visualization, Writing – review & editing. **Jinyu Li:** Visualization, Writing – review & editing. **Jiazhou Chen:** Writing – review & editing. **Robin B. Gasser:** Writing – review & editing. **Zhiyuan Yuan:** Supervision, Writing – review & editing. **Jiangning Song:** Supervision, Project administration, Funding acquisition, Writing – review & editing. **Hongmin Cai:** Supervision, Project administration, Funding acquisition, Writing – review & editing. All authors have read and approved the final manuscript.

## Competing interests

The authors have declared no competing interests.

## Supplementary material

Supplementary material is available at *Genomics, Proteomics & Bioinformatics* online (https://doi.org/10.1093/gpbjnl/qzaf128).

## Supplementary Material

qzaf128_Supplementary_Data
