# Supplementary material for: HyperSTAR: Unveiling Tissue Structure and Tumor Microenvironment from Spatial Omics by Hypergraph Learning
Source: Genomics Proteomics Bioinformatics. 2025 Dec 26;24(1):qzaf128. doi: 10.1093/gpbjnl/qzaf128 (PMC13317993; doi:10.1093/gpbjnl/qzaf128)
Supplement: qzaf128_Supplementary_Data [file qzaf128_supplementary_data.zip › Supplementary material captions.docx]

**Supplementary material**

**Figure S1 Boxplots comparing the performance of different methods across four metrics: ARI, NMI, Moran’s I, and Geary’s C**

Each box represents the interquartile range (IQR) of the values for each method, with the median indicated by the line inside the box.

**Figure S2 The result of Scanpy on DLPFC dataset sections 151507–151510**

**Figure S3 The result of Scanpy on DLPFC dataset sections 151569–151672**

**Figure S4 The result of Scanpy on DLPFC dataset sections 151573–151676**

**Figure S5 The result of SpaGCN on DLPFC dataset sections 151507–151510**

**Figure S6 The result of SpaGCN on DLPFC dataset sections 151569–151672**

**Figure S7 The result of SpaGCN on DLPFC dataset sections 151573–151676**

**Figure S8 The result of STAGATE on DLPFC dataset sections 151507–151510**

**Figure S9 The result of STAGATE on DLPFC dataset sections 151569–151672**

**Figure S10 The result of STAGATE on DLPFC dataset sections 151573–151676**

**Figure S11 The result of BayesSpace on DLPFC dataset sections 151507–151510**

**Figure S12 The result of BayesSpace on DLPFC dataset sections 151569–151672**

**Figure S13 The result of BayesSpace on DLPFC dataset sections 151573–151676**

**Figure S14 The result of HyperSTAR on DLPFC dataset sections 151507–151510**

**Figure S15 The result of HyperSTAR on DLPFC dataset sections 151569–151672**

**Figure S16 The result of HyperSTAR on DLPFC dataset sections 151573–151676**

**Figure S17 Performance of HyperSTAR under multiple runs**

1. The average Adjusted Rand Index (ARI) across 12 slices of the DLPFC dataset, with 10 independent runs for each slice. **B.** ARI values obtained from 10 independent runs of HyperSTAR for each slice in the DLPFC dataset. The error bars represent the standard deviation of the ARI values across the 10 runs.

**Figure S18 Analysis of Denoising Effects in Spatial Transcriptomics Data**

1. Annotation and HyperSTAR clustering results for DLPFC slice 151673. **B.** comparison of marker gene expression before and after denoising with HyperSTAR. **C.** Allen reference annotations and HyperSTAR clustering for the mouse olfactory bulb. **D.** Raw and HyperSTAR-denoised expression of marker genes alongside corresponding *In Situ* Hybridization (ISH) images from the Allen Brain Atlas.

**Figure S19 Identification of Spatially Variable Genes (SVGs) using HyperSTAR in the Marmoset Cerebellum**

1. Upper panel shows the expert annotations of the cerebellar sections, while the lower panel displays the clustering results obtained from HyperSTAR. **B.** Visualization of the top Highly Variable Genes (HVGs) for each cluster identified by HyperSTAR, after applying sc.tl.rank_genes_groups().

**Figure S20 HyperSTAR reveals different layers at finer resolutions of the Stereo-seq mouse olfactory bulb**

Spatial visualization of ACO, GR, LPL, and MI identified by HyperSTAR.

**Figure S21 HyperSTAR reveals different layers at finer resolutions of the Stereo-seq mouse olfactory bulb**

Spatial visualization of OPL, GL, and ONL identified by HyperSTAR.

**Figure S22 HyperSTAR achieves robust performance across different resolutions on Stereo-seq data**

1. Reference from the Allen Brain Atlas. **B.** Spatial visualization of results obtained by Scanpy, STAGATE, and HyperSTAR across four different resolutions (Single cell, bin 20, bin 50, and bin 100).

**Figure S23 HyperSTAR achieves robust performance across different resolutions on Stereo-seq data**

**A.** Reference from the Allen Brain Atlas. **B.** Results obtained by HyperSTAR on bin 20 mouse olfactory bulb data. c Spatial visualization of different layers identified by HyperSTAR and their corresponding marker gene expression.

**Figure S24 HyperSTAR reveals layers of adult axolotl brain**

1. Annotation of adult axolotl brain data. **B.** Spatial visualization of results obtained by Scanpy, STAGATE, and HyperSTAR.

**Figure S25 Comparative Performance of Clustering Methods on breast cancer and osmFISH dataset**

1. Manual annotations and metrics comparison for the breast cancer dataset, highlighting Moran’s I and Geary’s C. **B.** Spatial visualization of clustering results from four methods (Scanpy, STAGATE, GraphST, and HyperSTAR) on the breast cancer dataset. **C.** Manual annotations alongside spatial visualizations of clustering outcomes for the osmFISH dataset using the same four methods. **D.** Metrics comparison for the osmFISH dataset, including ARI and NMI.

**Figure S26 The survival analysis of IDC DEGs**

**Figure S27 HyerSTAR is applicable to various spatial omics and different platforms**

1. Manual annotation and HyperSTAR identification of somatosensory cortex from osmFISH. **B.** The mouse brain reference from Allen Atlas. **C.** The spatial result of HyperSTAR on a mouse brain section from Xenium. **D.** The spatial result and UMAP plot by HyperSTAR on a mouse brain section from MERFISH. E. Manual annotation and HyperSTAR identification of a Barrett’s esophagus section from CODEX. **F.** The PanCK distribution and HyperSTAR identification of a NSCLC section from CosMx.

**Figure S28 The data quality in mouse olfactory bulb data of Stereoseq and SlideSeqv2**

**A.** Data quality of Stereoseq data. **B.** Data quality of SlideSeqV2 data.

**Figure S29 The joint clustering results**

1. UMAP plots depicting the data distribution before integration (left) and after Harmony integration (right). **B.** Comparison of HyperSTAR and STAligner based on spatial autocorrelation metrics, Moran’s I and Geary’s C. **C.** UMAP plots of the joint embedding (left) alongside spatial visualization of clustering results (right) for HyperSTAR (lower) and STAligner (upper).
