# Supplementary material for: HyperSTAR: Unveiling Tissue Structure and Tumor Microenvironment from Spatial Omics by Hypergraph Learning
Source: Genomics Proteomics Bioinformatics. 2025 Dec 26;24(1):qzaf128. doi: 10.1093/gpbjnl/qzaf128 (PMC13317993; doi:10.1093/gpbjnl/qzaf128)
Supplement: qzaf128_Supplementary_Data [file qzaf128_supplementary_data.zip › Supplementary figure S1-S29.pdf]

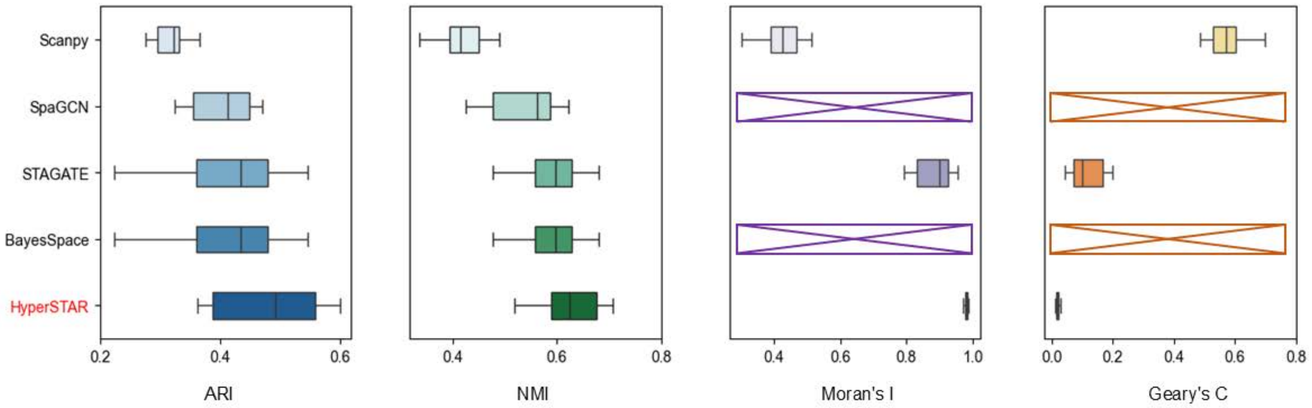

Scanpy
SpaGCN
STAGATE
BayesSpace
HyperSTAR
0.2
0.4
0.6
ARI
0.4
0.6
0.8
NMI
0.4
0.6
0.8
1.0
Moran's I
0.0
0.2
0.4
0.6
0.8
Geary's C

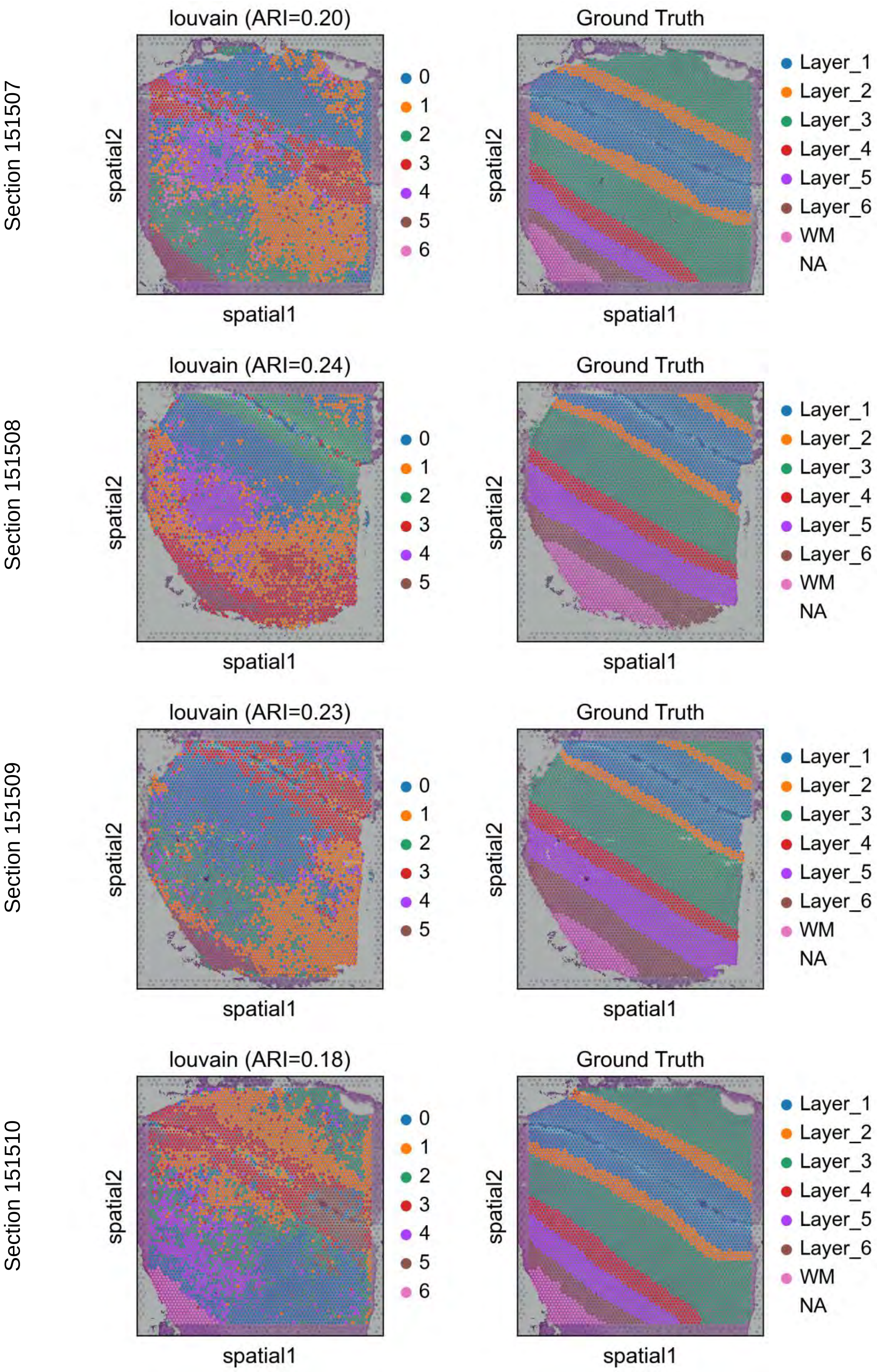

Section 151507
louvain (ARI=0.20)
Ground Truth
Section 151508
louvain (ARI=0.24)
Ground Truth
Section 151509
louvain (ARI=0.23)
Ground Truth
Section 151510
louvain (ARI=0.18)
Ground Truth
spatial1
spatial2
0
1
2
3
4
5
6
Layer_1
Layer_2
Layer_3
Layer_4
Layer_5
Layer_6
WM
NA

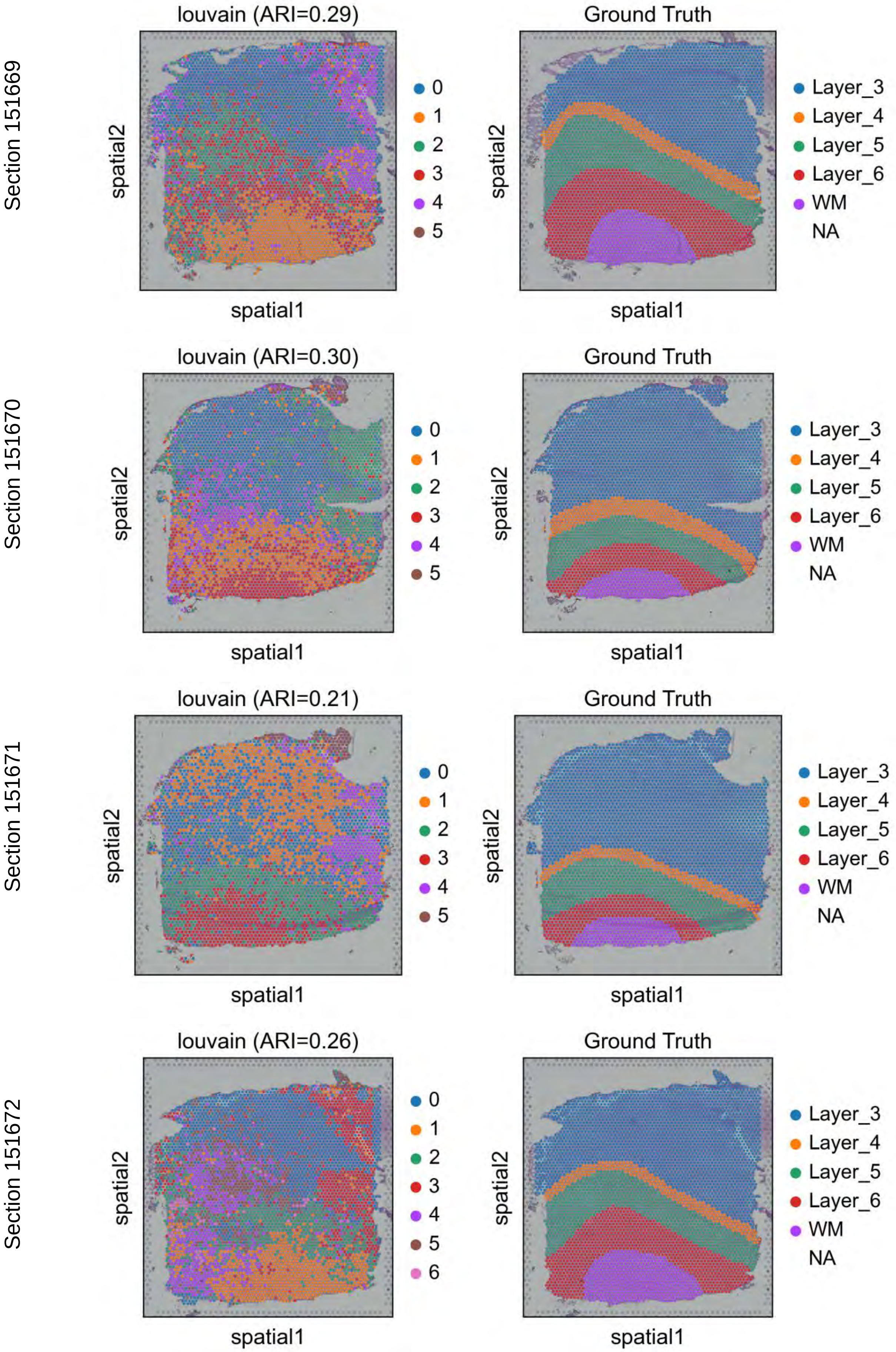
Section 151669
louvain (ARI=0.29)
Ground Truth
Section 151670
louvain (ARI=0.30)
Ground Truth
Section 151671
louvain (ARI=0.21)
Ground Truth
Section 151672
louvain (ARI=0.26)
Ground Truth
spatial1
spatial2
0
1
2
3
4
5
6
Layer_3
Layer_4
Layer_5
Layer_6
WM
NA

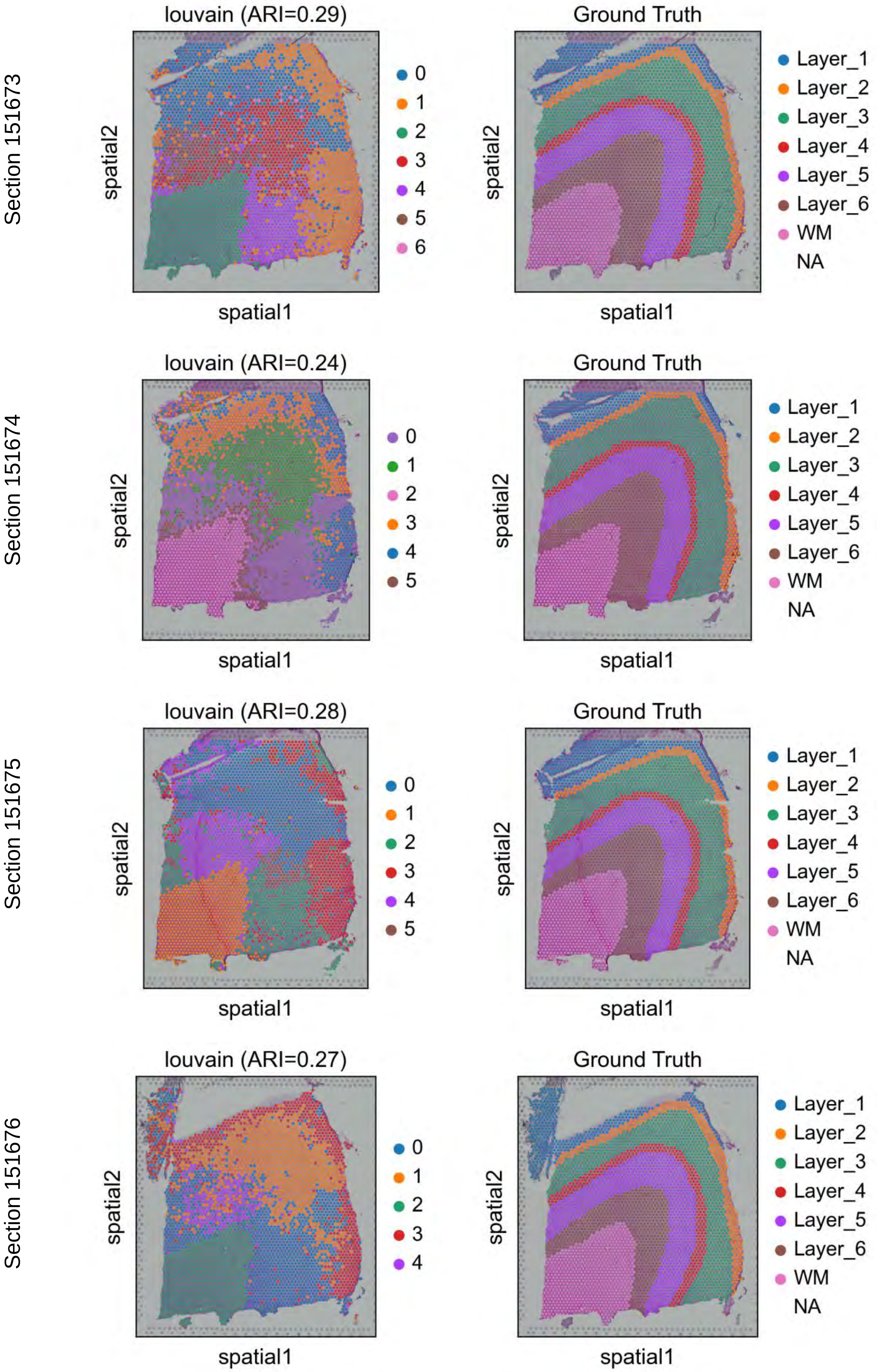
Section 151673
louvain (ARI=0.29)
Ground Truth
Section 151674
louvain (ARI=0.24)
Ground Truth
Section 151675
louvain (ARI=0.28)
Ground Truth
Section 151676
louvain (ARI=0.27)
Ground Truth
spatial1
spatial2
0
1
2
3
4
5
6
Layer_1
Layer_2
Layer_3
Layer_4
Layer_5
Layer_6
WM
NA

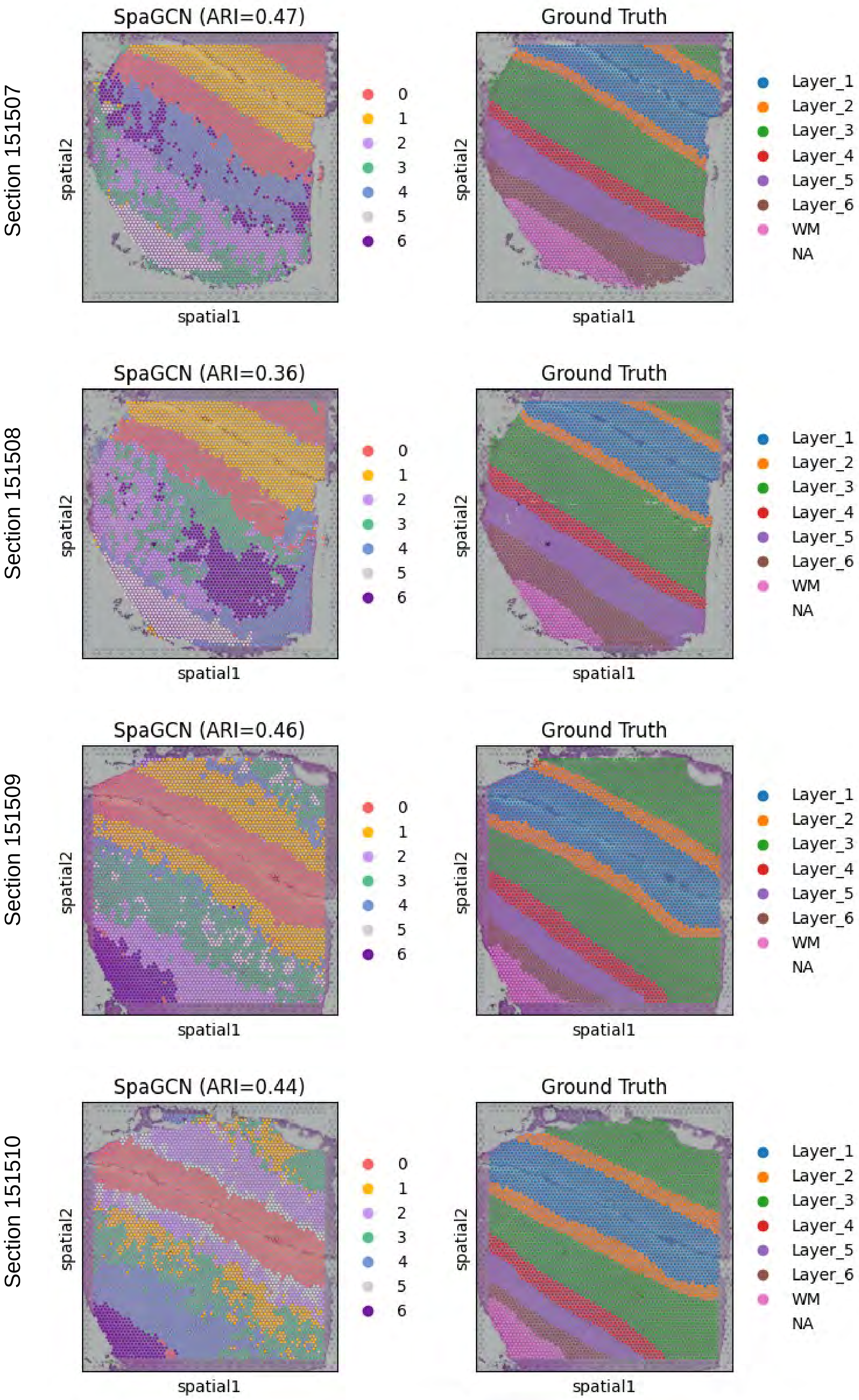
Section 151507
SpaGCN (ARI=0.47)
Ground Truth
Section 151508
SpaGCN (ARI=0.36)
Ground Truth
Section 151509
SpaGCN (ARI=0.46)
Ground Truth
Section 151510
SpaGCN (ARI=0.44)
Ground Truth
spatial1
spatial2
0
1
2
3
4
5
6
Layer_1
Layer_2
Layer_3
Layer_4
Layer_5
Layer_6
WM
NA

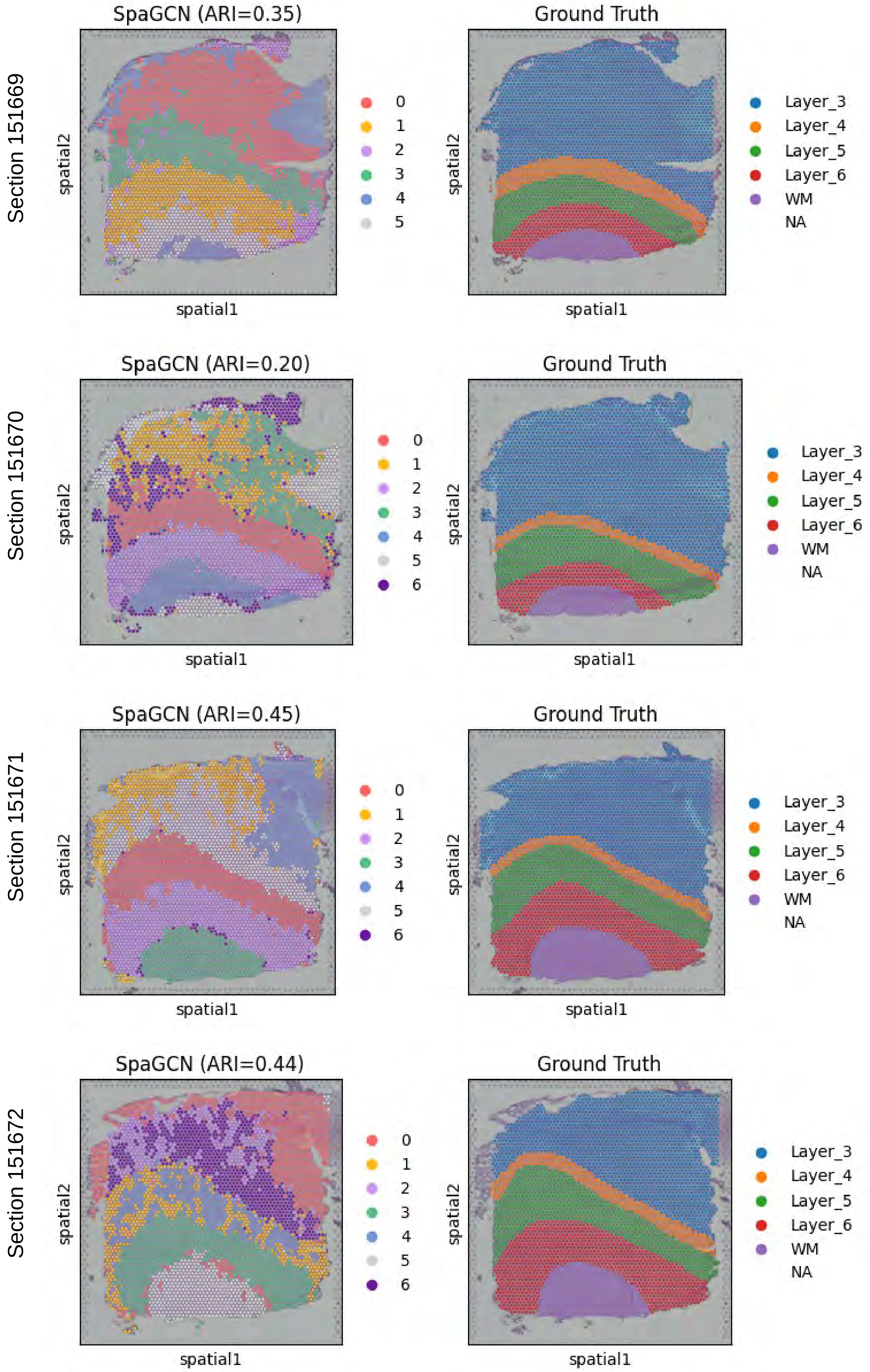

Section 151669
SpaGCN (ARI=0.35)
Ground Truth
Section 151670
SpaGCN (ARI=0.20)
Ground Truth
Section 151671
SpaGCN (ARI=0.45)
Ground Truth
Section 151672
SpaGCN (ARI=0.44)
Ground Truth
spatial1
spatial2
0
1
2
3
4
5
6
Layer_3
Layer_4
Layer_5
Layer_6
WM
NA

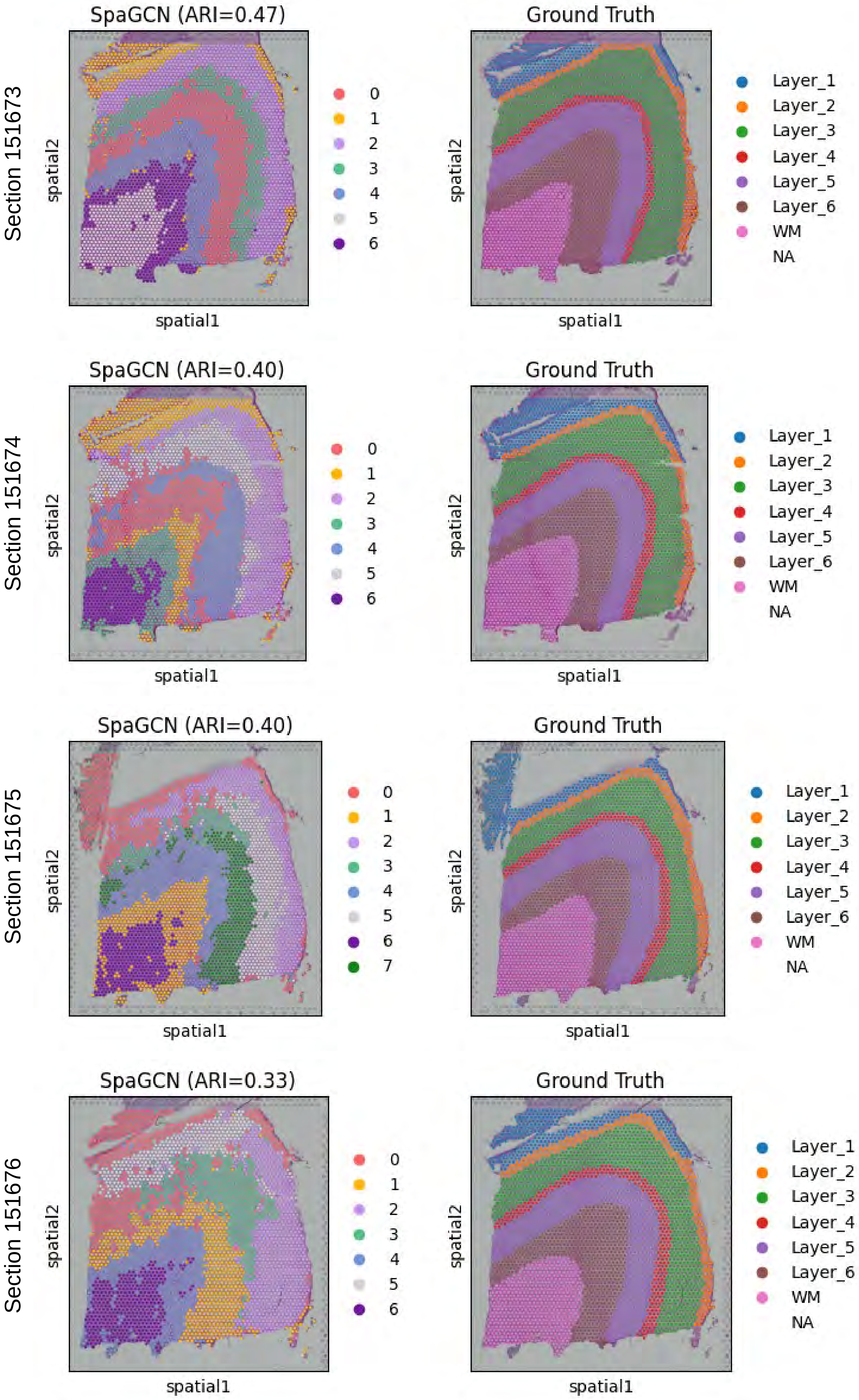
Section 151673
SpaGCN (ARI=0.47)
Ground Truth
Section 151674
SpaGCN (ARI=0.40)
Ground Truth
Section 151675
SpaGCN (ARI=0.40)
Ground Truth
Section 151676
SpaGCN (ARI=0.33)
Ground Truth
spatial1
spatial2
0
1
2
3
4
5
6
7
Layer_1
Layer_2
Layer_3
Layer_4
Layer_5
Layer_6
WM
NA

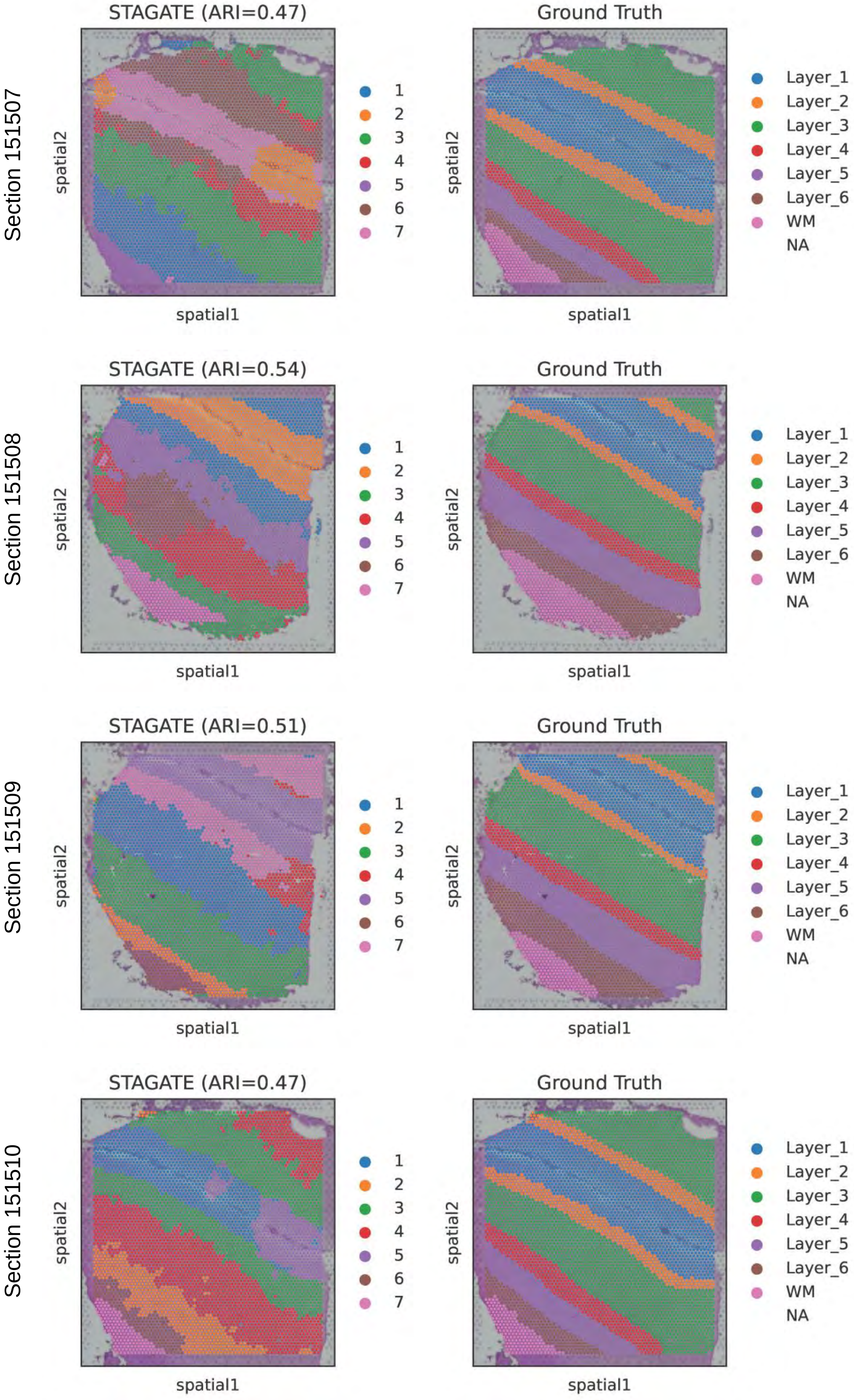
Section 151507
STAGATE (ARI=0.47)
Ground Truth
Section 151508
STAGATE (ARI=0.54)
Ground Truth
Section 151509
STAGATE (ARI=0.51)
Ground Truth
Section 151510
STAGATE (ARI=0.47)
Ground Truth
spatial1
spatial2
1
2
3
4
5
6
7
Layer_1
Layer_2
Layer_3
Layer_4
Layer_5
Layer_6
WM
NA

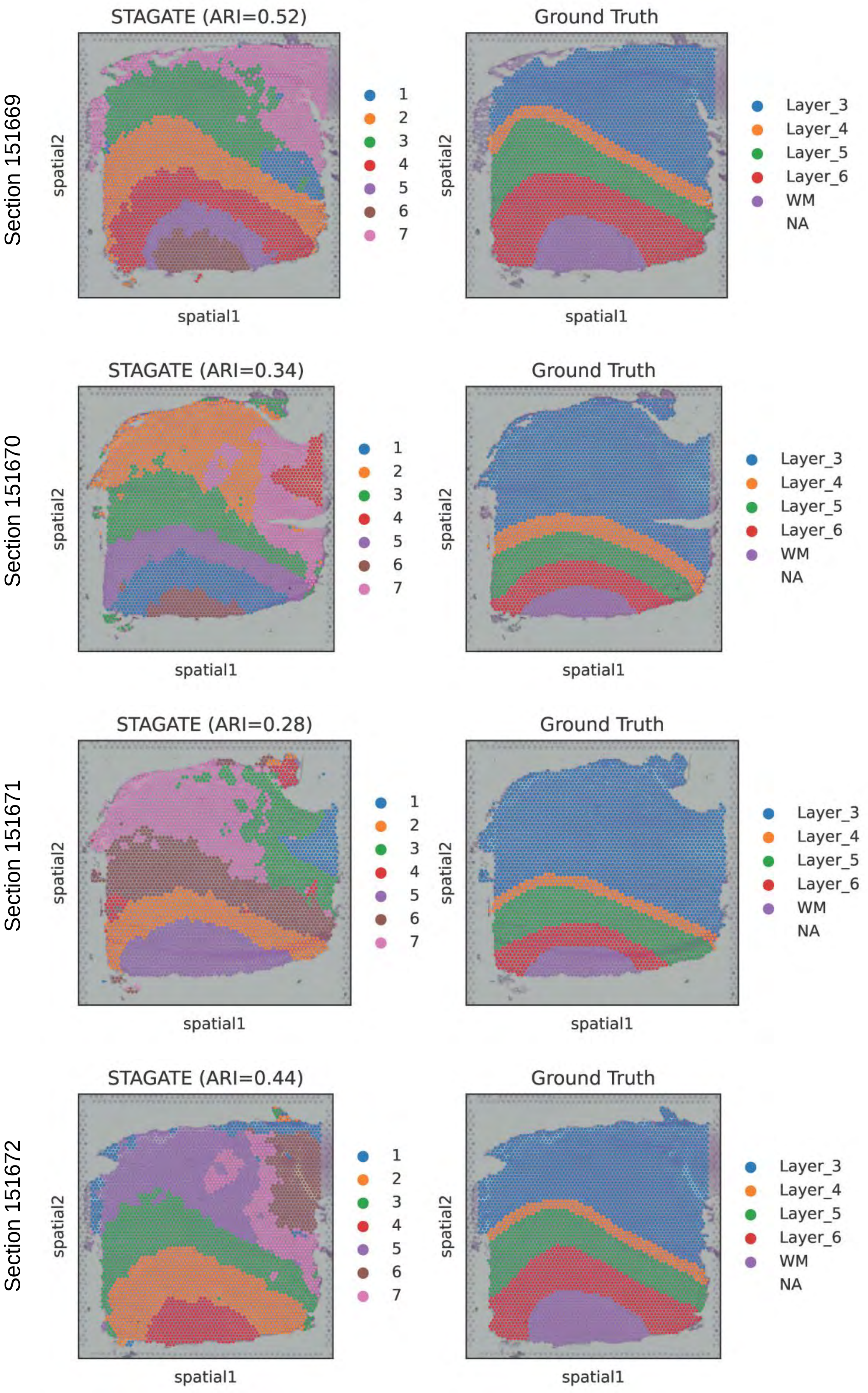

Section 151669
STAGATE (ARI=0.52)
Ground Truth
Section 151670
STAGATE (ARI=0.34)
Ground Truth
Section 151671
STAGATE (ARI=0.28)
Ground Truth
Section 151672
STAGATE (ARI=0.44)
Ground Truth
spatial1
spatial2
1
2
3
4
5
6
7
Layer_3
Layer_4
Layer_5
Layer_6
WM
NA

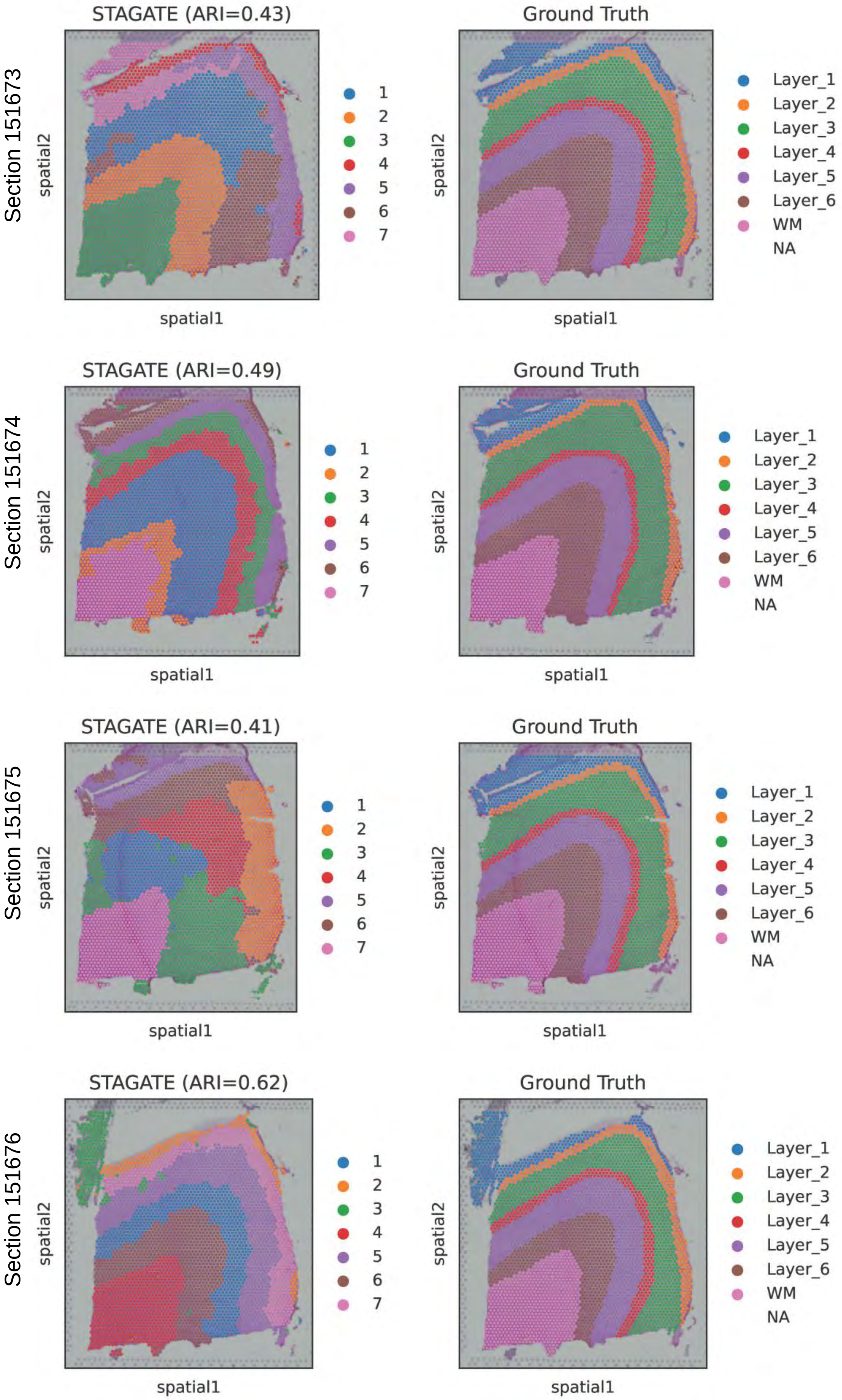
Section 151673
STAGATE (ARI=0.43)
spatial2
spatial1
1
2
3
4
5
6
7
Ground Truth
spatial2
spatial1
Layer_1
Layer_2
Layer_3
Layer_4
Layer_5
Layer_6
WM
NA
Section 151674
STAGATE (ARI=0.49)
spatial2
spatial1
1
2
3
4
5
6
7
Ground Truth
spatial2
spatial1
Layer_1
Layer_2
Layer_3
Layer_4
Layer_5
Layer_6
WM
NA
Section 151675
STAGATE (ARI=0.41)
spatial2
spatial1
1
2
3
4
5
6
7
Ground Truth
spatial2
spatial1
Layer_1
Layer_2
Layer_3
Layer_4
Layer_5
Layer_6
WM
NA
Section 151676
STAGATE (ARI=0.62)
spatial2
spatial1
1
2
3
4
5
6
7
Ground Truth
spatial2
spatial1
Layer_1
Layer_2
Layer_3
Layer_4
Layer_5
Layer_6
WM
NA

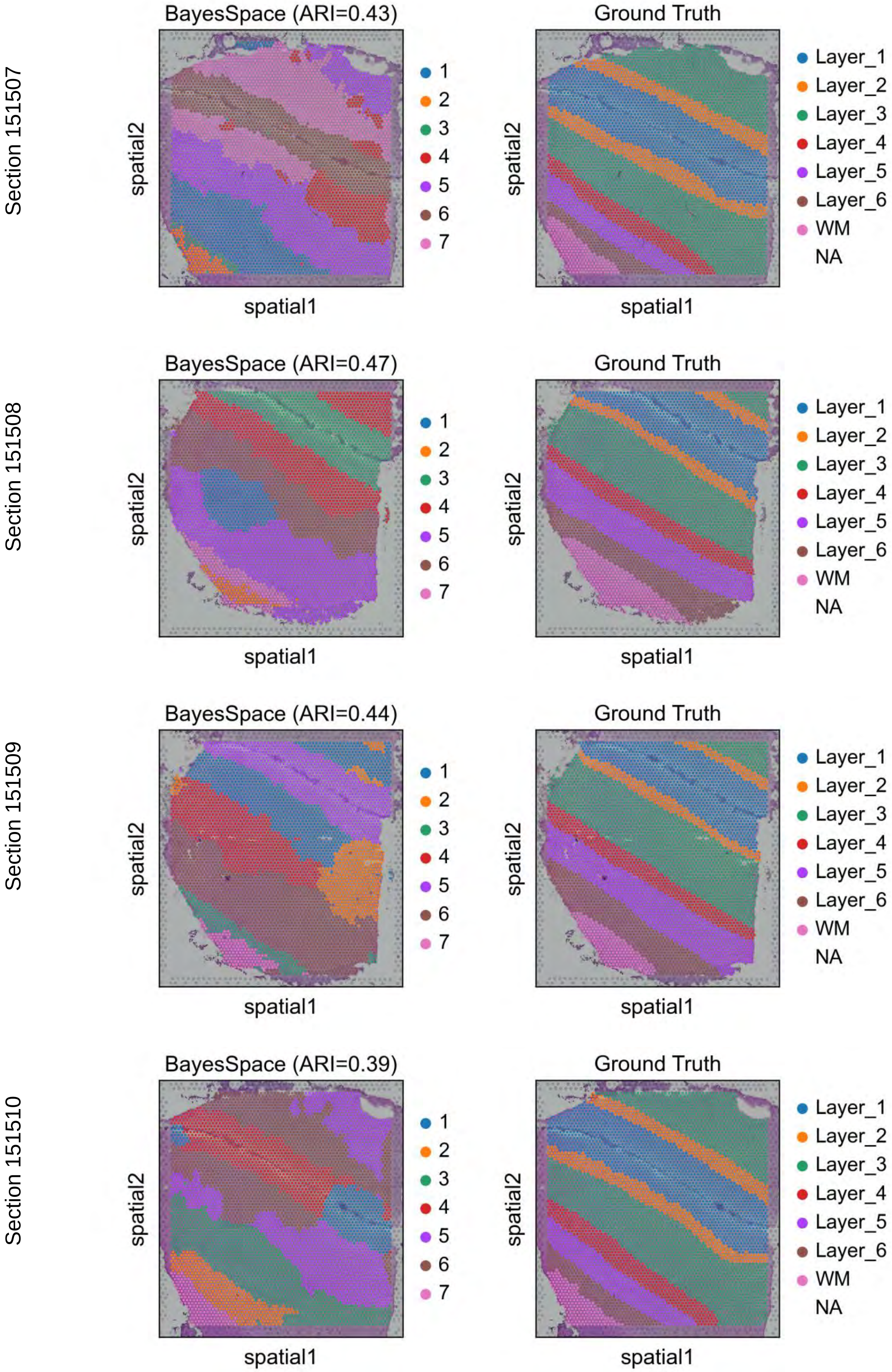
Section 151507
BayesSpace (ARI=0.43)
Ground Truth
Section 151508
BayesSpace (ARI=0.47)
Ground Truth
Section 151509
BayesSpace (ARI=0.44)
Ground Truth
Section 151510
BayesSpace (ARI=0.39)
Ground Truth
spatial1
spatial2
1
2
3
4
5
6
7
Layer_1
Layer_2
Layer_3
Layer_4
Layer_5
Layer_6
WM
NA

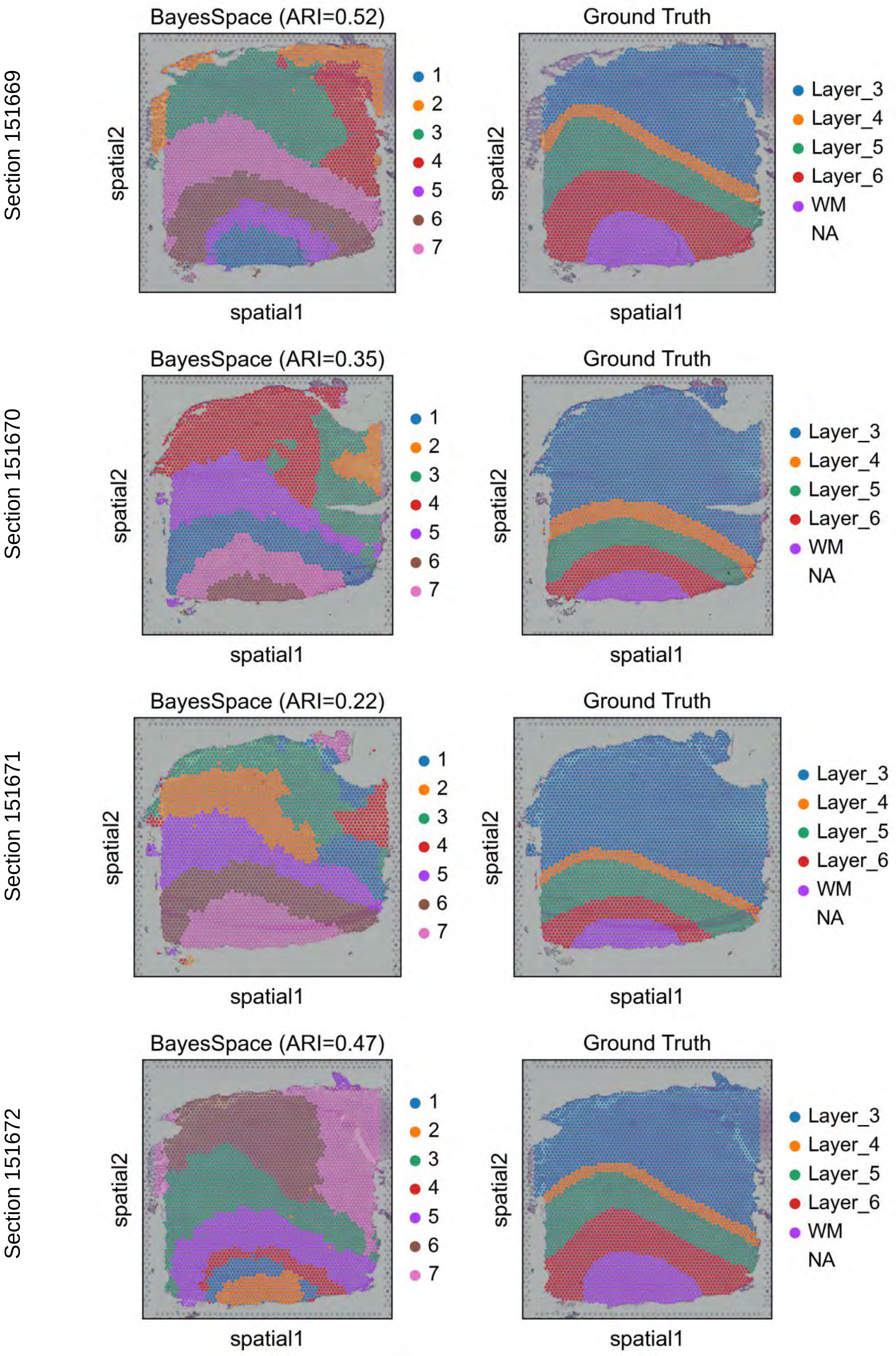
Section 151669
BayesSpace (ARI=0.52)
Ground Truth
Section 151670
BayesSpace (ARI=0.35)
Ground Truth
Section 151671
BayesSpace (ARI=0.22)
Ground Truth
Section 151672
BayesSpace (ARI=0.47)
Ground Truth
spatial1
spatial2
1
2
3
4
5
6
7
Layer_3
Layer_4
Layer_5
Layer_6
WM
NA

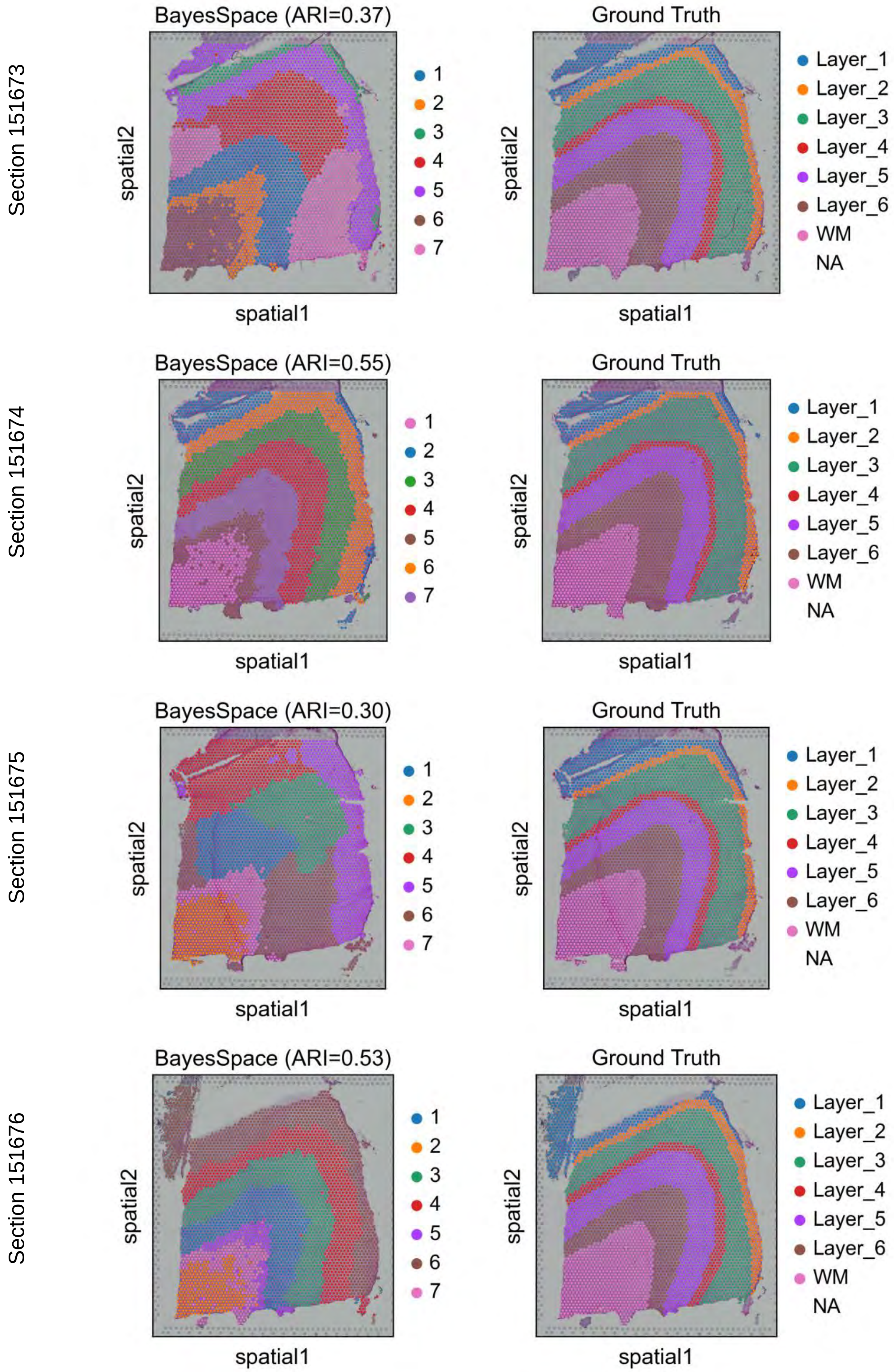
Section 151673
BayesSpace (ARI=0.37)
Ground Truth
Section 151674
BayesSpace (ARI=0.55)
Ground Truth
Section 151675
BayesSpace (ARI=0.30)
Ground Truth
Section 151676
BayesSpace (ARI=0.53)
Ground Truth
spatial1
spatial2
1
2
3
4
5
6
7
Layer_1
Layer_2
Layer_3
Layer_4
Layer_5
Layer_6
WM
NA

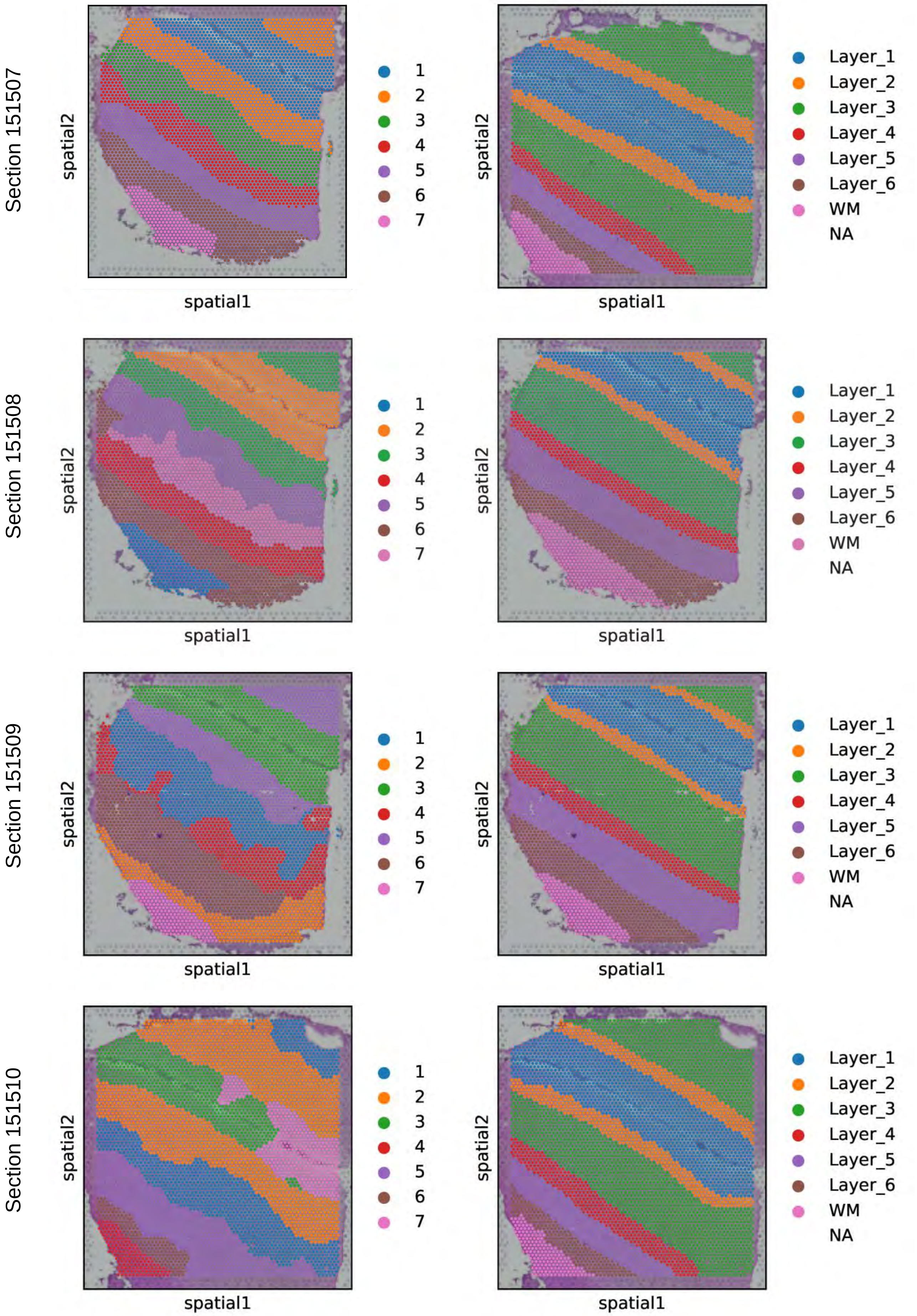
Section 151507
spatial2
spatial1
1
2
3
4
5
6
7
Layer_1
Layer_2
Layer_3
Layer_4
Layer_5
Layer_6
WM
NA
Section 151508
Section 151509
Section 151510

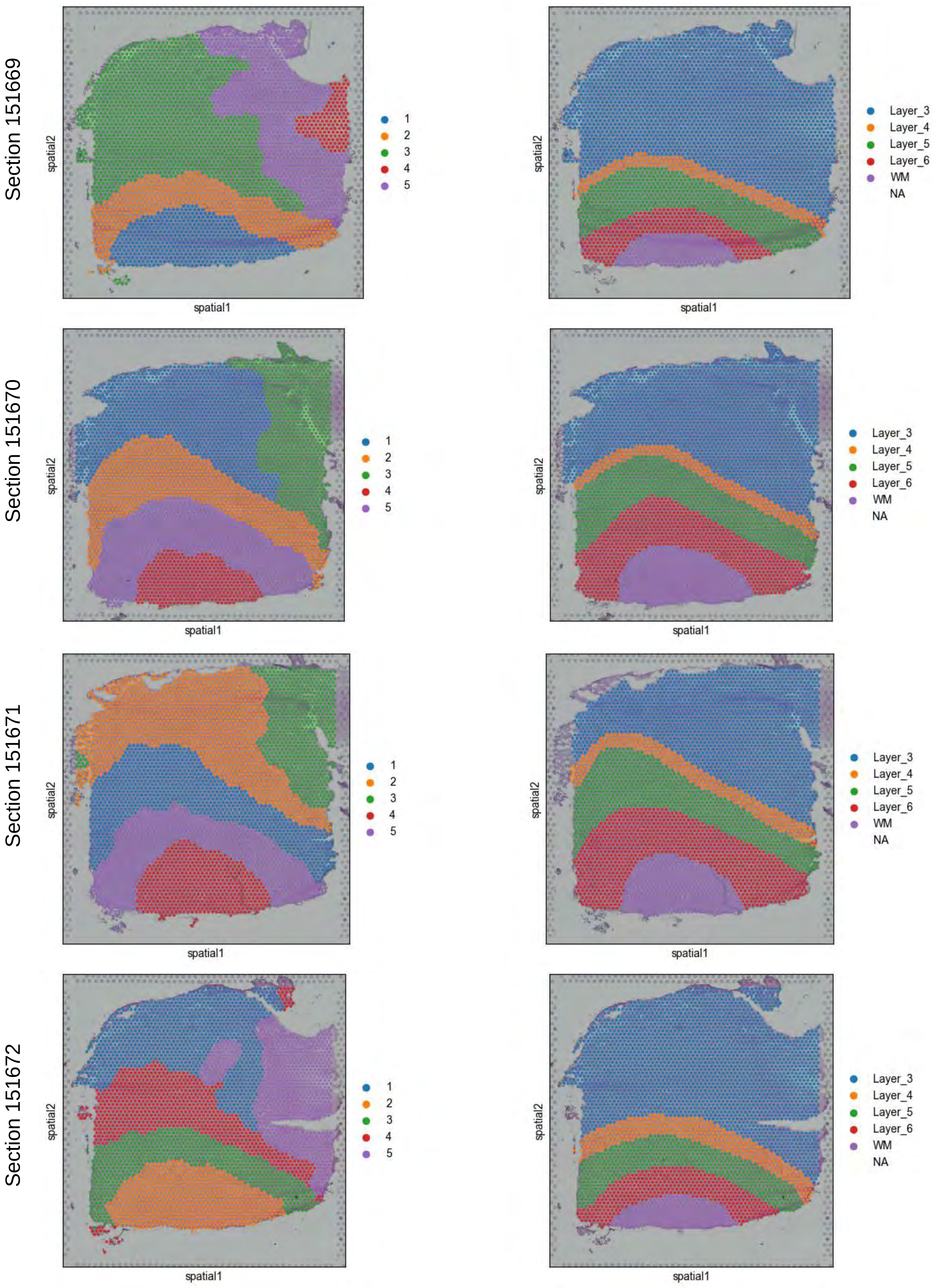

Section 151669
Section 151670
Section 151671
Section 151672
spatial1
spatial2
1
2
3
4
5
Layer_3
Layer_4
Layer_5
Layer_6
WM
NA

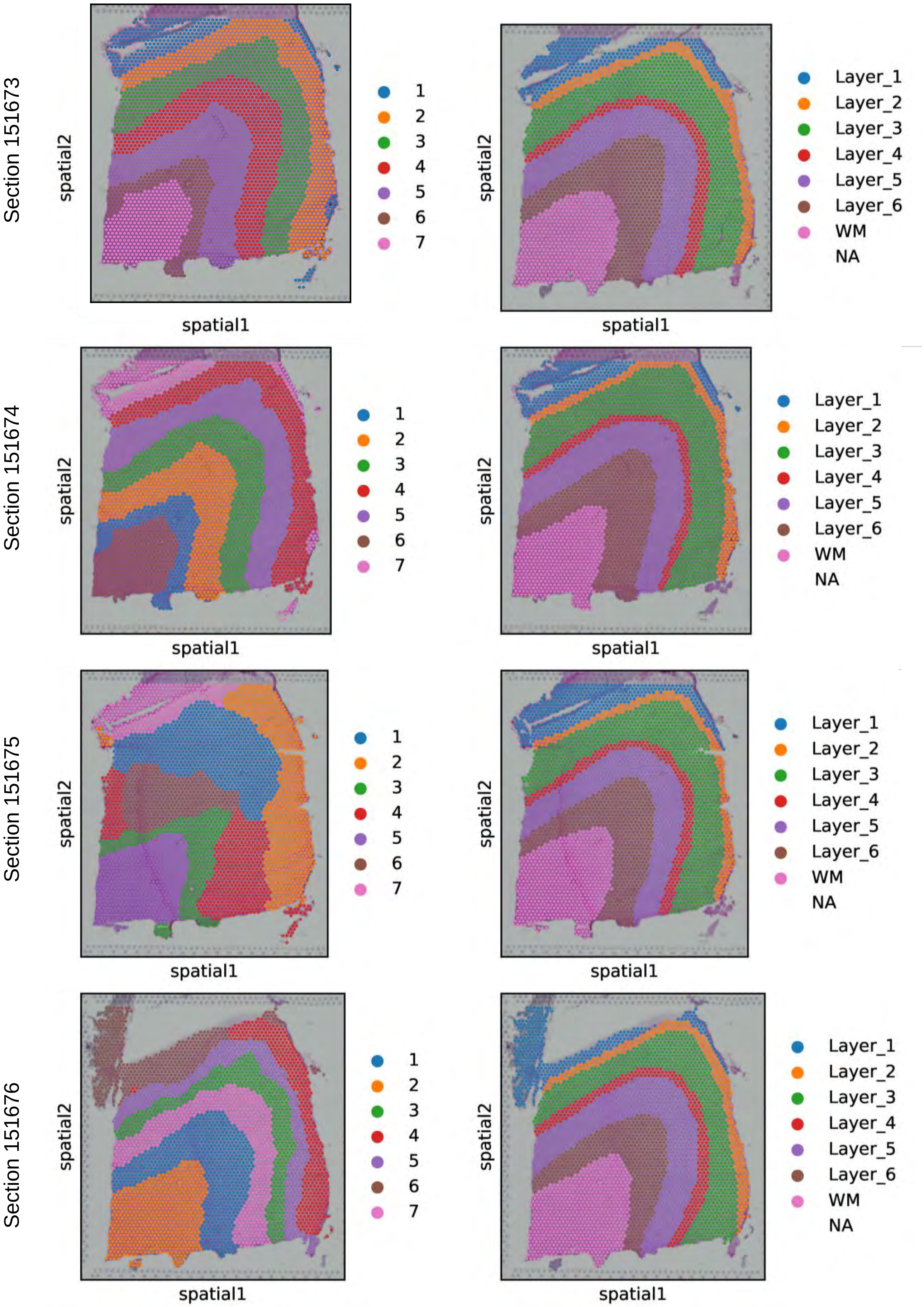
Section 151673
Section 151674
Section 151675
Section 151676
spatial1
spatial2
1
2
3
4
5
6
7
Layer_1
Layer_2
Layer_3
Layer_4
Layer_5
Layer_6
WM
NA

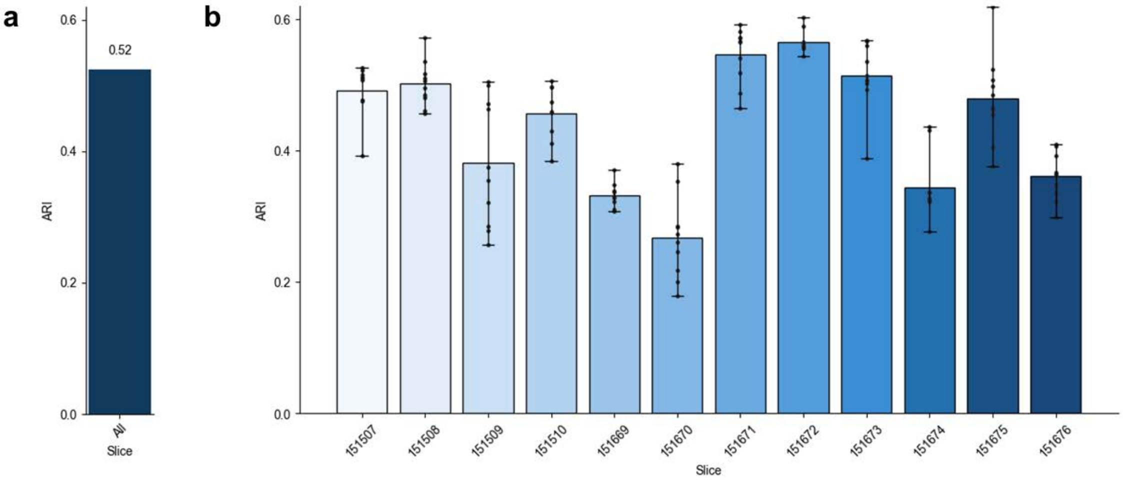
a
b
0.52
ARI
Slice
All
151507
151508
151509
151510
151669
151670
151671
151672
151673
151674
151675
151676
0.0
0.2
0.4
0.6

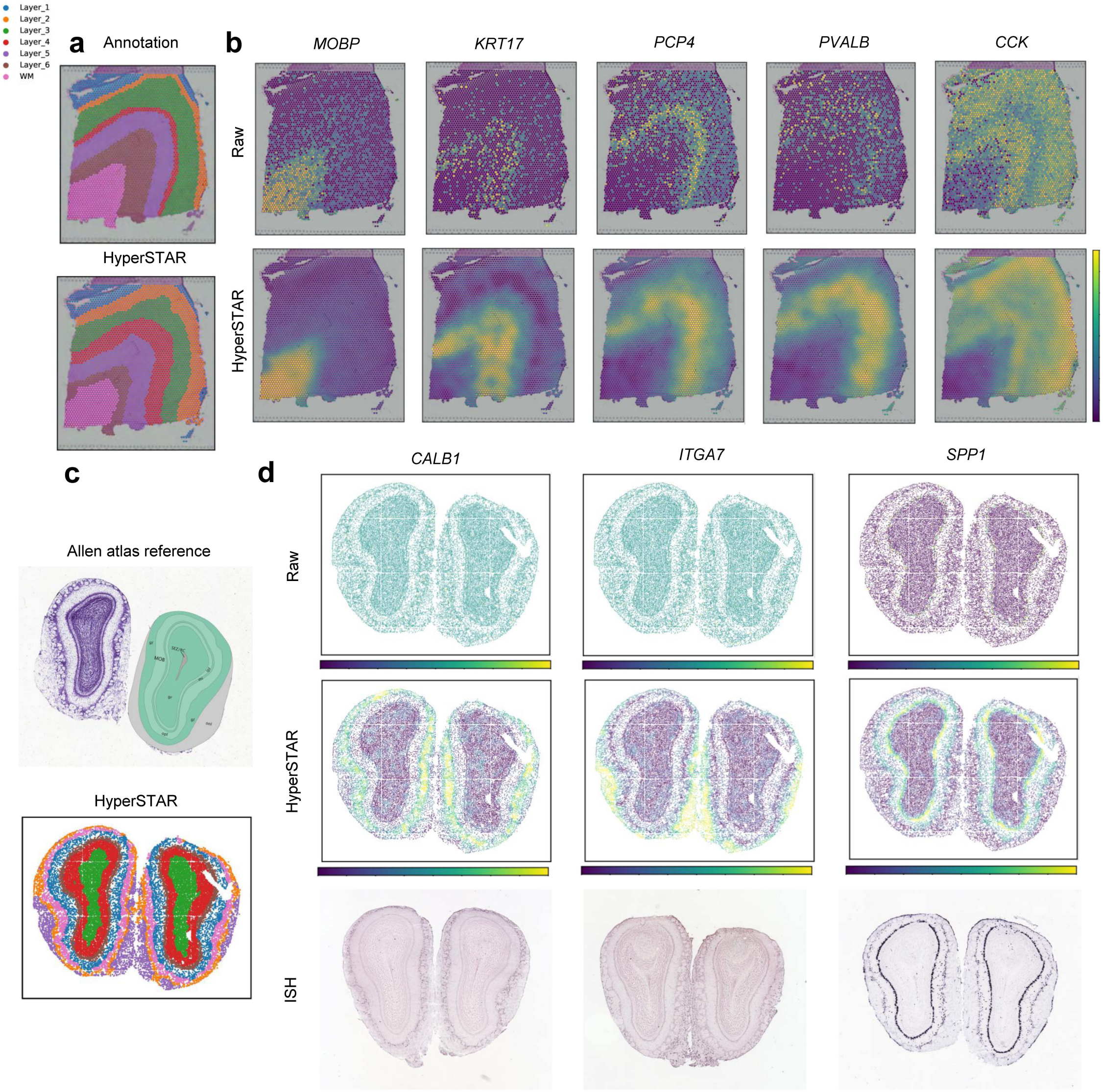

Layer_1
Layer_2
Layer_3
Layer_4
Layer_5
Layer_6
WM
a
Annotation
HyperSTAR
b
MOBP
KRT17
PCP4
PVALB
CCK
Raw
HyperSTAR
c
Allen atlas reference
HyperSTAR
d
CALB1
ITGA7
SPP1
Raw
HyperSTAR
ISH

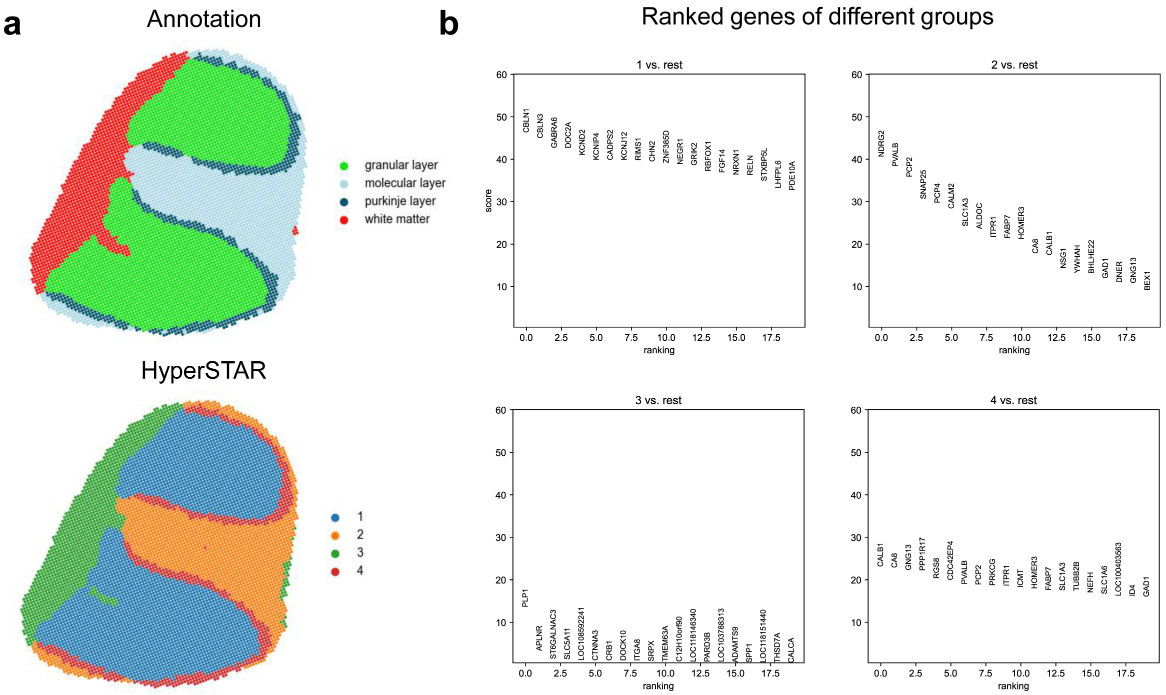

a
Annotation
granular layer
molecular layer
purkinje layer
white matter
HyperSTAR
1
2
3
4
b
Ranked genes of different groups
1 vs. rest
CBLN1
CBLN3
GABRA6
DOC2A
KCND2
KCNIP4
CADPS2
KCNJ12
RIMS1
CHN2
ZNF385D
NEGR1
GRIK2
RBFOX1
FGF14
NRXN1
RELN
STXBP5L
LHFPL6
PDE10A
2 vs. rest
NDRG2
PVALB
PCP2
SNAP25
PCP4
CALM2
SLC1A3
ALDOC
ITPR1
FABP7
HOMER3
CA8
CALB1
NSG1
YWHAH
BHLHE22
GAD1
DNER
GNG13
BEX1
3 vs. rest
PLP1
APLNR
ST6GALNAC3
SLC5A11
LOC108592241
CTNNA3
CRB1
DOCK10
ITGA8
SRPX
TMEM63A
C12H10orf90
LOC118146340
PARD3B
LOC103788313
ADAMTS9
SPP1
LOC118151440
THSD7A
CALCA
4 vs. rest
CALB1
CA8
GNG13
PPP1R17
RGS8
CDC42EP4
PVALB
PCP2
PRKCG
ITPR1
ICMT
HOMER3
FABP7
SLC1A3
TUBB2B
NEFH
SLC1A6
LOC100403563
ID4
GAD1
score
ranking

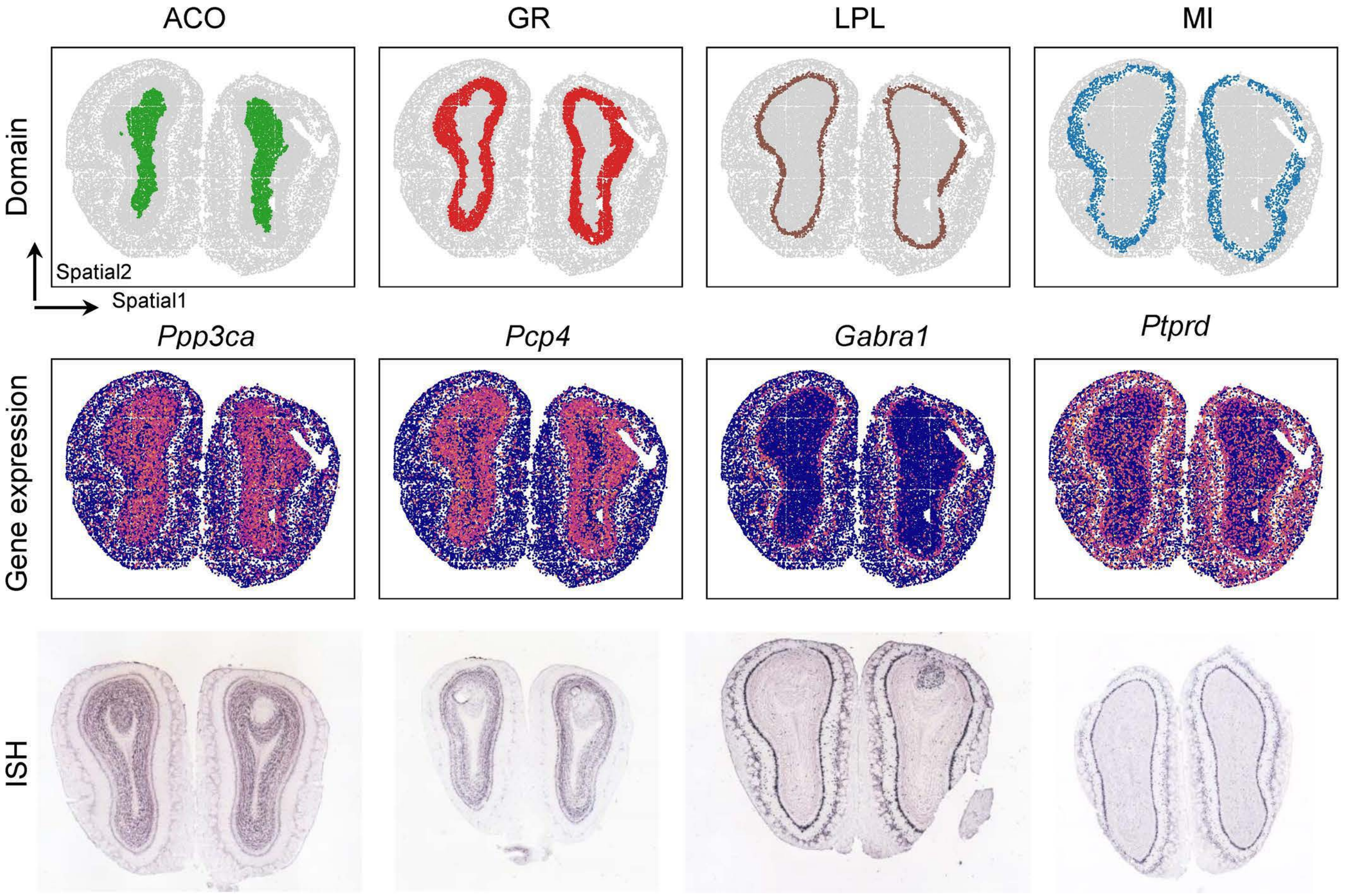

ACO
GR
LPL
MI
Domain
Spatial2
Spatial1
Ppp3ca
Pcp4
Gabra1
Ptprd
Gene expression
ISH

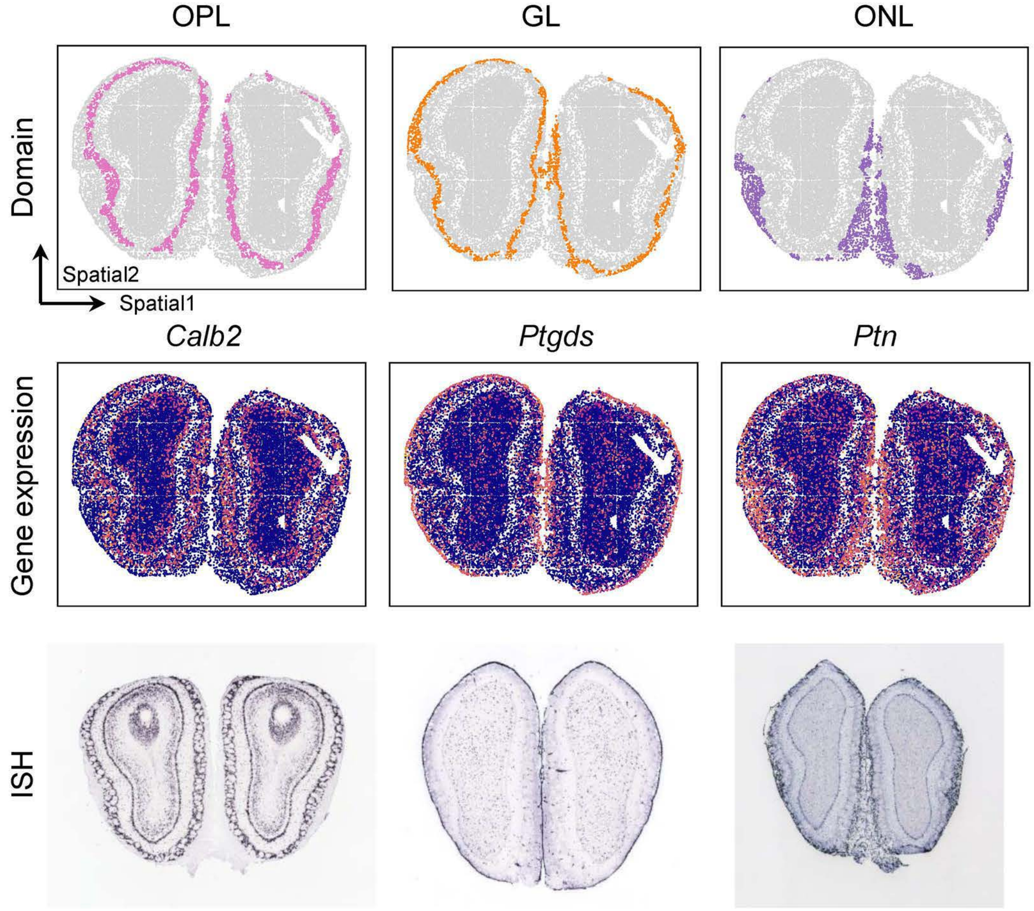
OPL
GL
ONL
Domain
Spatial2
Spatial1
Calb2
Ptgds
Ptn
Gene expression
ISH

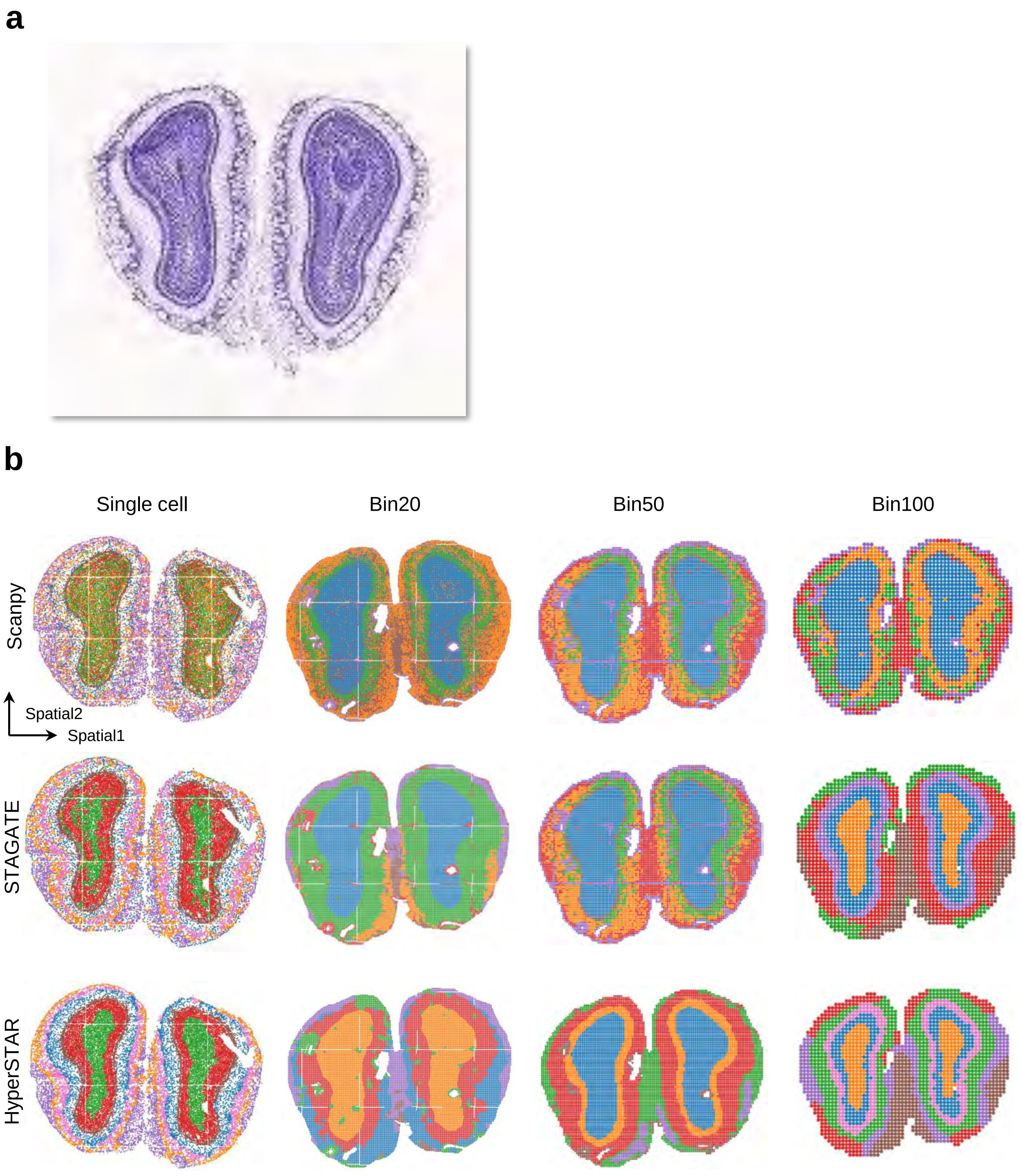
a
b
Single cell
Bin20
Bin50
Bin100
Scanpy
STAGATE
HyperSTAR
Spatial2
Spatial1

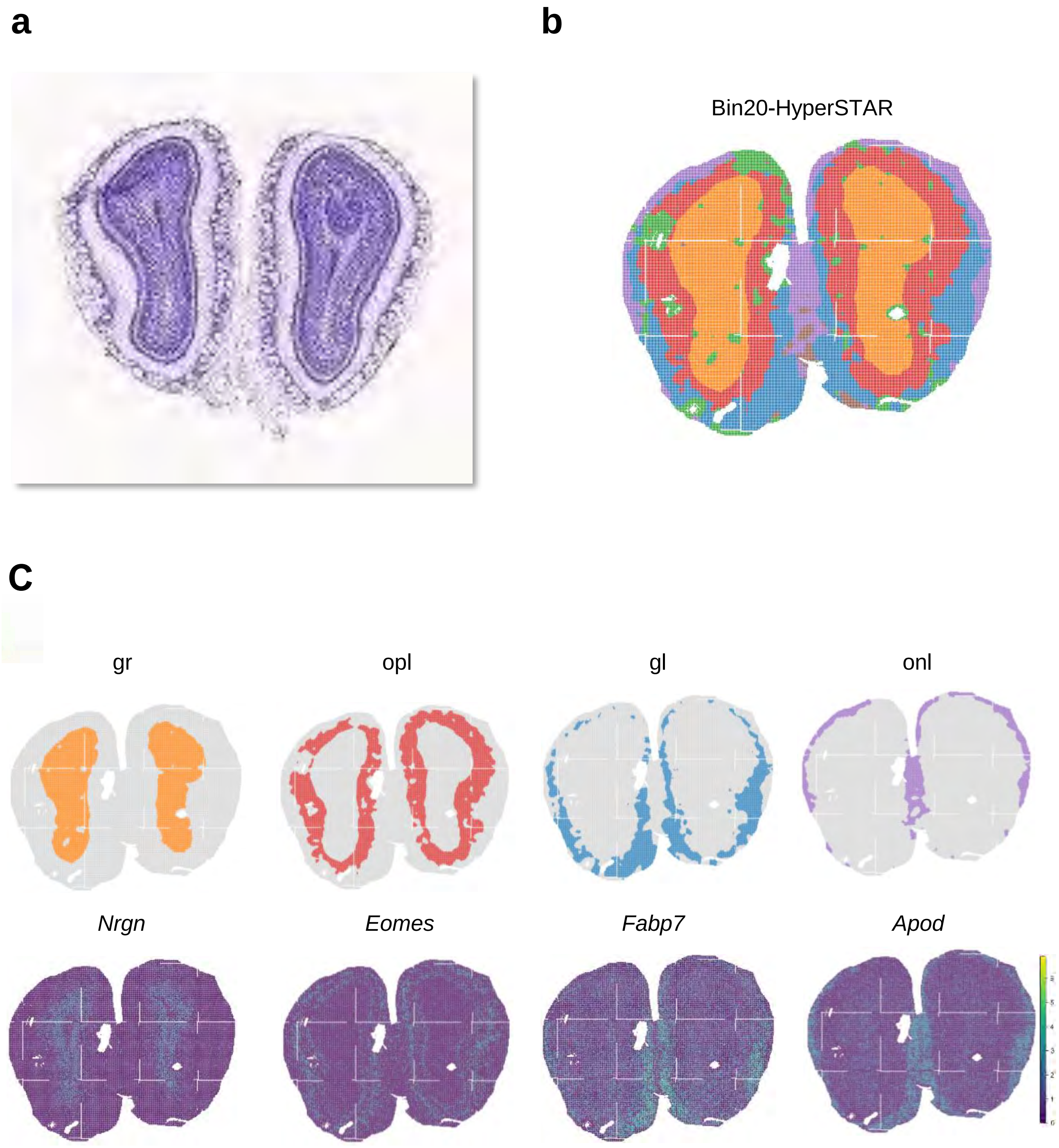
a
b
Bin20-HyperSTAR
c
gr
opl
gl
onl
Nrgn
Eomes
Fabp7
Apod

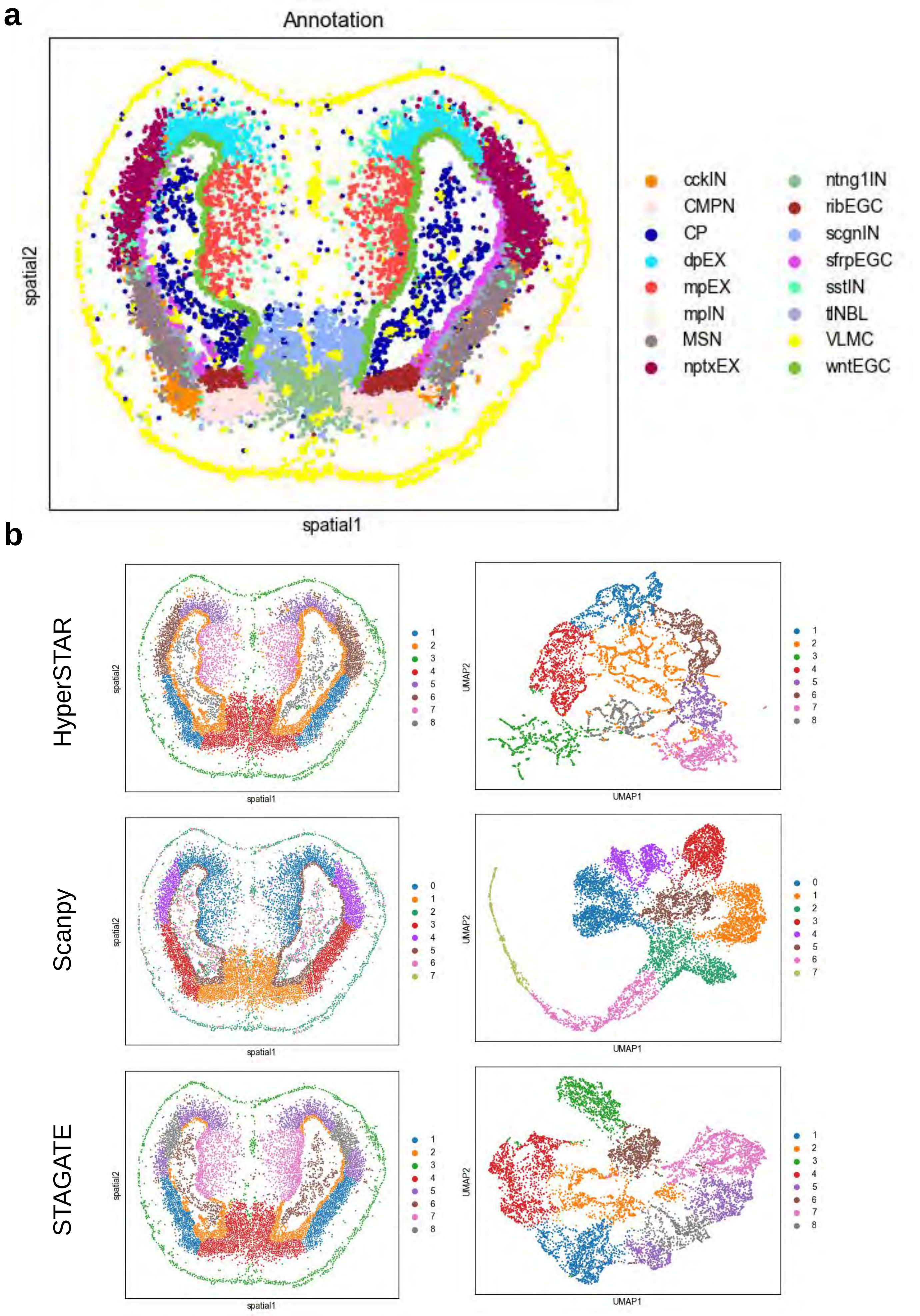

a
Annotation
spatial2
spatial1
cckIN
CMPN
CP
dpEX
mpEX
mpIN
MSN
nptxEX
ntng1IN
ribEGC
scgnIN
sfrpEGC
sstIN
tlNBL
VLMC
wntEGC
b
HyperSTAR
Scanpy
STAGATE
spatial2
spatial1
UMAP2
UMAP1
1
2
3
4
5
6
7
8
0

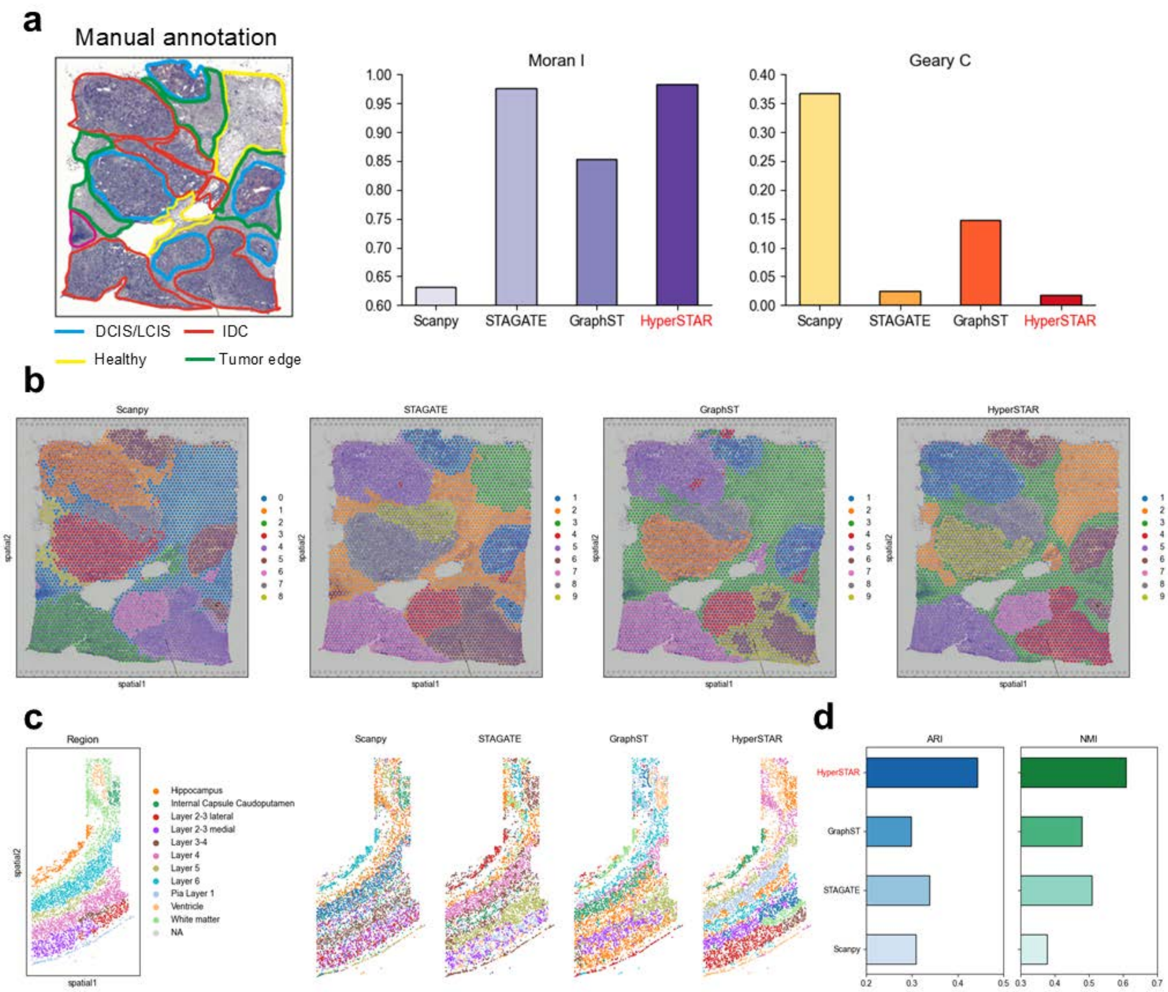

a
Manual annotation
DCIS/LCIS
IDC
Healthy
Tumor edge
Moran I
Geary C
Scanpy
STAGATE
GraphST
HyperSTAR
b
Scanpy
STAGATE
GraphST
HyperSTAR
spatial1
spatial2
c
Region
Hippocampus
Internal Capsule Caudoputamen
Layer 2-3 lateral
Layer 2-3 medial
Layer 3-4
Layer 4
Layer 5
Layer 6
Pia Layer 1
Ventricle
White matter
NA
d
ARI
NMI

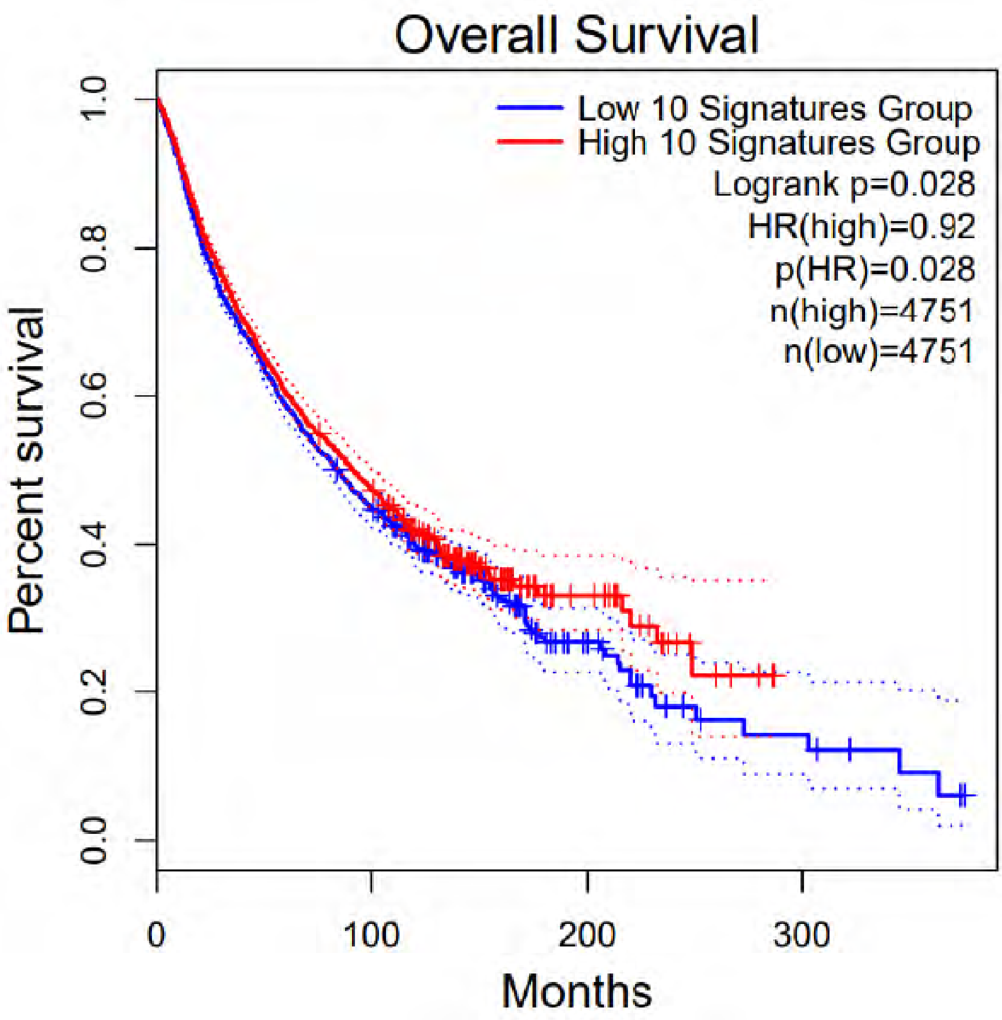

Overall Survival
Low 10 Signatures Group
High 10 Signatures Group
Logrank p=0.028
HR(high)=0.92
p(HR)=0.028
n(high)=4751
n(low)=4751
Percent survival
1.0
0.8
0.6
0.4
0.2
0.0
0
100
200
300
Months

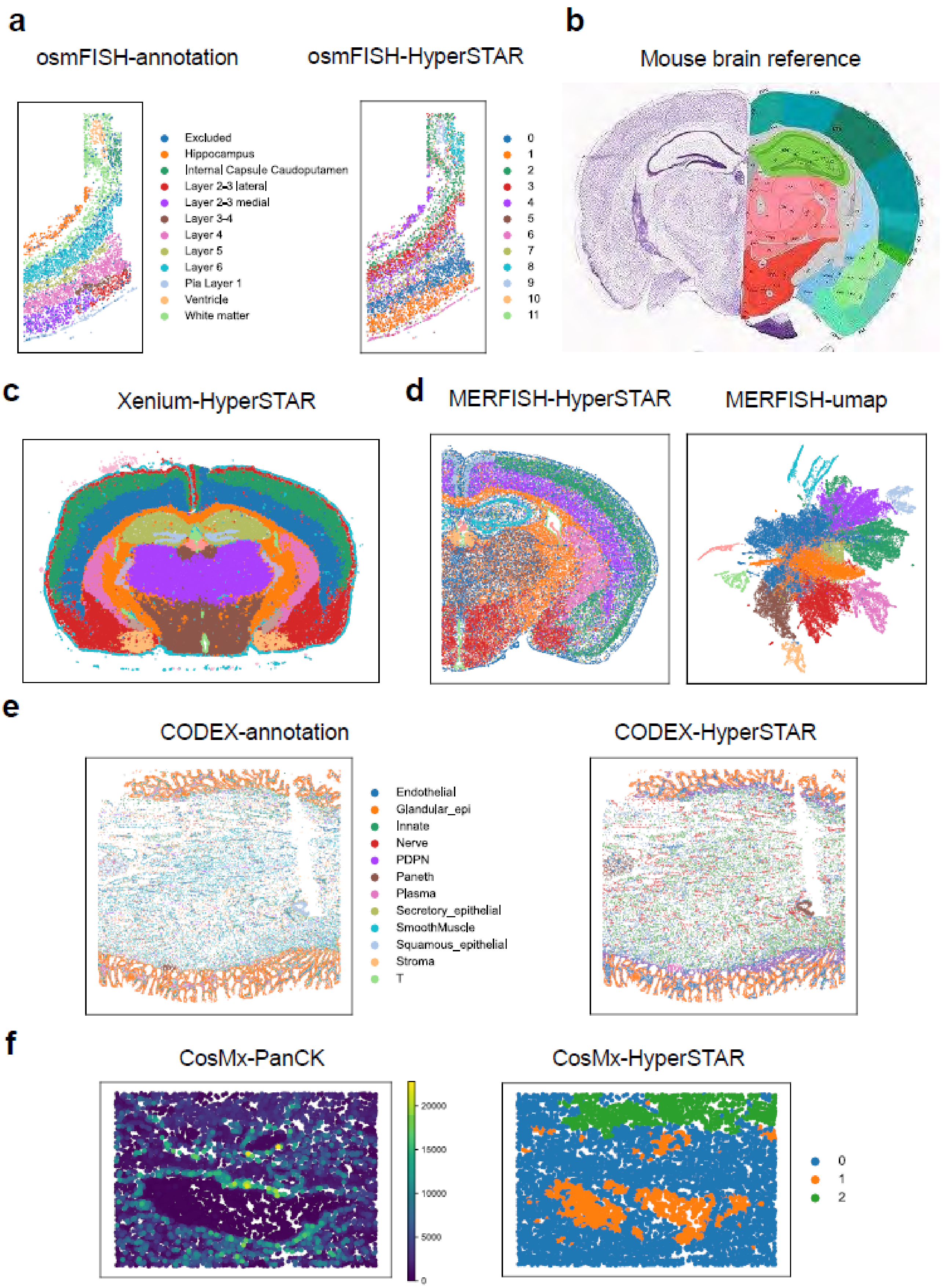
a
osmFISH-annotation
osmFISH-HyperSTAR
Excluded
Hippocampus
Internal Capsule Caudoputamen
Layer 2-3 lateral
Layer 2-3 medial
Layer 3-4
Layer 4
Layer 5
Layer 6
Pia Layer 1
Ventricle
White matter
0
1
2
3
4
5
6
7
8
9
10
11
b
Mouse brain reference
c
Xenium-HyperSTAR
d
MERFISH-HyperSTAR
MERFISH-umap
e
CODEX-annotation
CODEX-HyperSTAR
Endothelial
Glandular_epi
Innate
Nerve
PDPN
Paneth
Plasma
Secretory_epithelial
SmoothMuscle
Squamous_epithelial
Stroma
T
f
CosMx-PanCK
CosMx-HyperSTAR
20000
15000
10000
5000
0
0
1
2

a

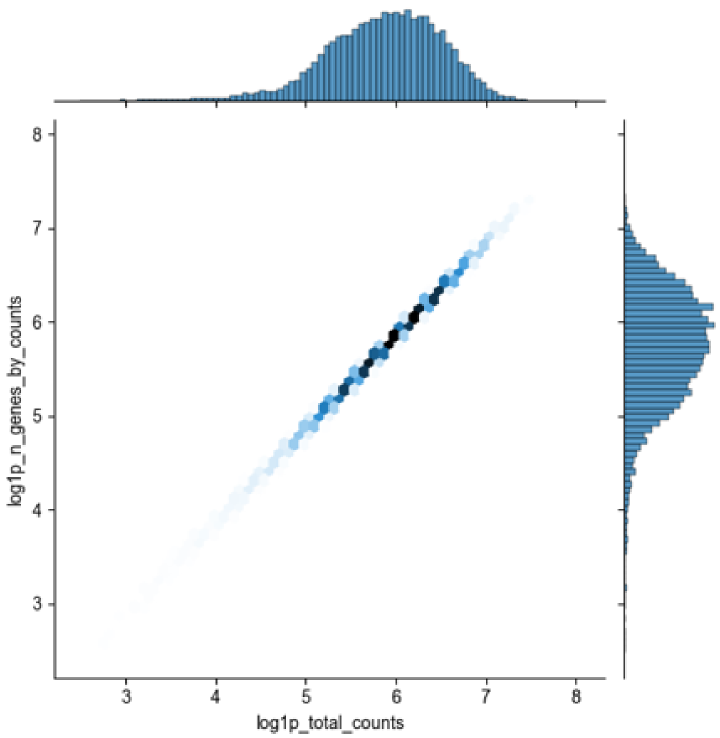
log1p_n_genes_by_counts
log1p_total_counts
3 4 5 6 7 8

b

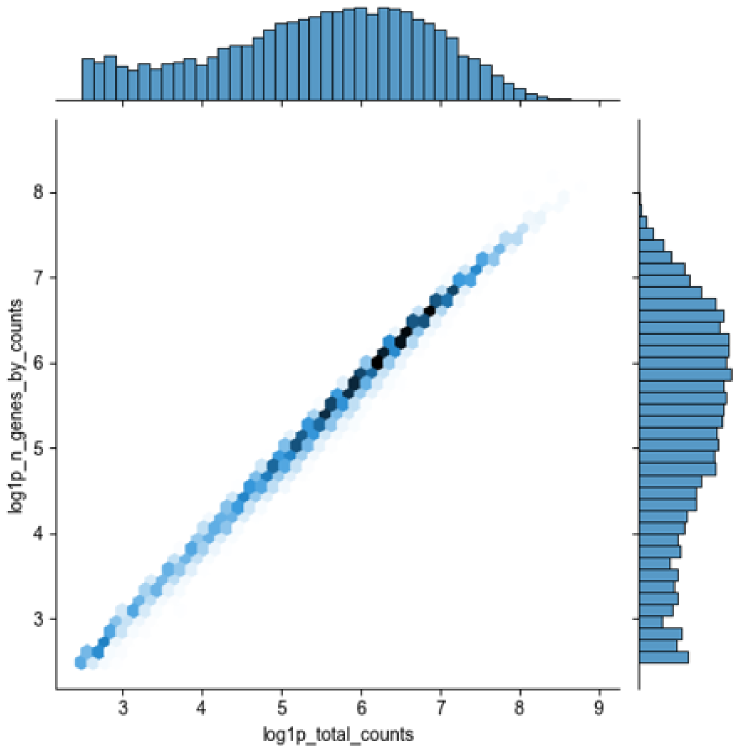
log1p_n_genes_by_counts
log1p_total_counts
3 4 5 6 7 8 9

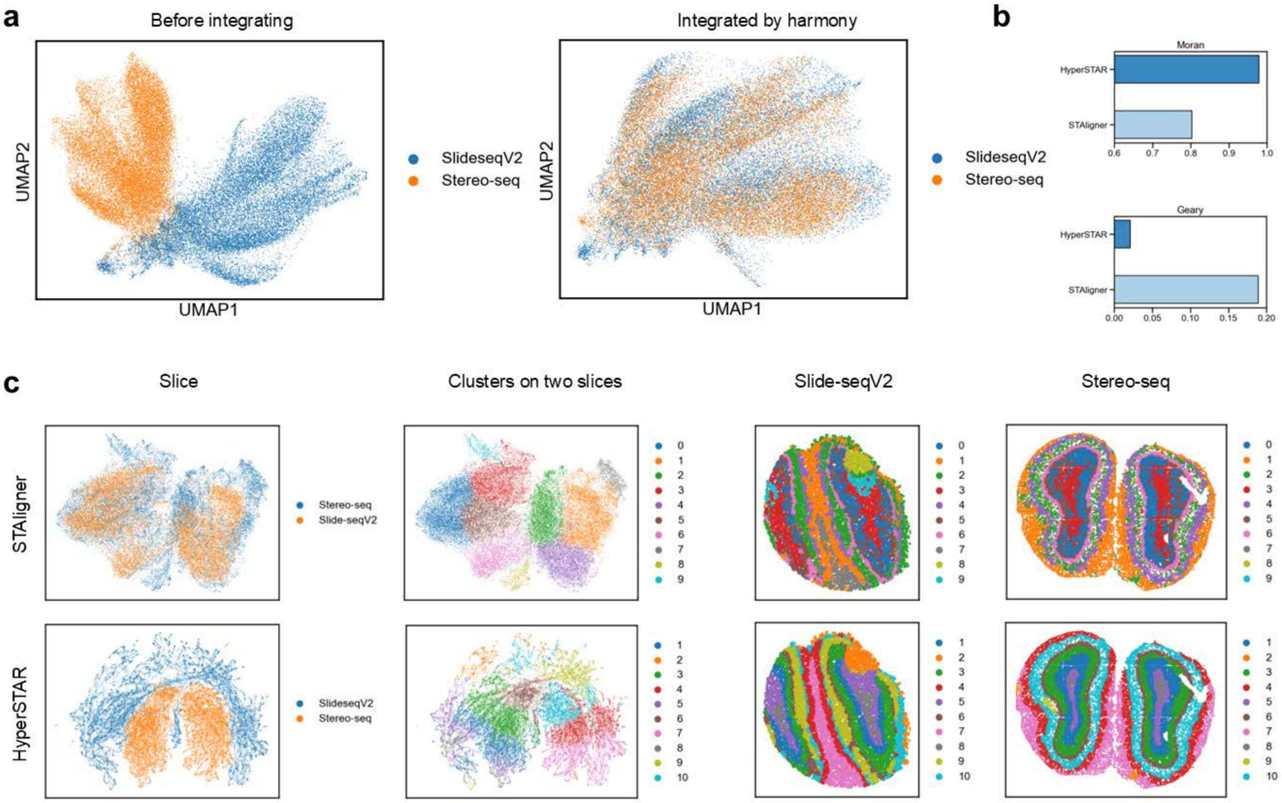

a
Before integrating
Integrated by harmony
UMAP1
UMAP2
SlideseqV2
Stereo-seq
b
Moran
Geary
HyperSTAR
STAligner
c
Slice
Clusters on two slices
Slide-seqV2
Stereo-seq
STAligner
HyperSTAR
Stereo-seq
Slide-seqV2
SlideseqV2
